# Leveraging historical trials to predict Fusarium head blight resistance in spring wheat breeding programs

**DOI:** 10.1002/tpg2.20559

**Published:** 2025-02-06

**Authors:** Charlotte Brault, Emily J. Conley, Andrew J. Green, Karl D. Glover, Jason P. Cook, Harsimardeep S. Gill, Andrew C. Read, Jason D. Fiedler, James A. Anderson

**Affiliations:** ^1^ Department of Agronomy and Plant Genetics University of Minnesota St. Paul Minnesota USA; ^2^ Department of Plant Sciences North Dakota State University Fargo North Dakota USA; ^3^ Agronomy, Horticulture, and Plant Science Department South Dakota State University Brookings South Dakota USA; ^4^ Plant Sciences and Plant Pathology Department Montana State University Bozeman Montana USA; ^5^ USDA‐ARS Plant Science Research Unit St. Paul Minnesota USA; ^6^ USDA‐ARS Cereal Crops Research Unit Edward T. Schafer Agricultural Research Center Fargo North Dakota USA

## Abstract

Fusarium head blight (FHB) is a fungal disease posing a major threat to wheat production. Plant breeding that leverages genotyping is an effective method to improve the genetic resistance of cultivars. Started in 1995, the uniform regional scab nursery (URSN) consists of germplasm from several public breeding programs in the Northern US region. Its main objective is to showcase new sources of resistance and enable germplasm exchange among the cooperators; however, the data from the URSN have not been studied. Phenotypic and genotypic data from this nursery were gathered, as well as from two current breeding programs in the US Midwest. Genomic prediction on eight traits related to FHB and agronomic traits was applied, and the effects of statistical method, marker density, training set size, genetic structure, and genetic architecture of the trait were studied. Using the URSN population, reproducing kernel Hilbert space was the best method in various prediction settings, with an average accuracy of 0.63, marker density could be as low as 500 without decreasing the prediction accuracy, and training set optimization was useful for two traits. Furthermore, genotypic values were predicted in breeding programs using the URSN population as a training set with various prediction scenarios. Predicting unrelated populations led to a significant decrease in accuracy but with encouraging values for some traits and populations. Ultimately, when progressively decreasing the number of lines from breeding populations in the training set, the advantage of adding the URSN population was more pronounced, with an increase in accuracy up to 0.19.

## Core Ideas

- Genomic predictive ability ranged from 0.49 to 0.72 for predicting Fusarium head blight (FHB) and agronomical traits in wheat.
- Training set size had more impact on accuracy than marker density, which could be reduced to between 500 and 1000 markers.
- Training set optimization with a sparse selection index increased the accuracy of genomic prediction for two traits.
- Adding an unrelated population in the training set allows a reduction in phenotyping effort.

**Abbreviations**
BLUP: best linear unbiased predictor
DIS: disease index
DON: deoxynivalenol
FHB: Fusarium head blight
GP: genomic prediction
HD: heading date
INC: disease incidence
LASSO: least absolute shrinkage selection operator
MSU: Montana State University
mTWT: micro‐test weight
NDSU: North Dakota State University
PCA: principal component analysis
QTL: quantitative trait locus
RKHS: reproducing kernel Hilbert space
RR‐BLUP: ridge regression BLUP
SDSU: South Dakota State University
SEV: disease severity
SNP: single nucleotide polymorphism
SSI: sparse selection index
SW30: weight of 30 spikes
UMN: University of Minnesota
URN: Uniform Regional Nursery
URSN: Uniform Regional Scab Nreursery
VSK: visual scabby kernel

## 1 INTRODUCTION

Fusarium head blight (FHB, also called scab) has damaged wheat (*Triticum aestivum* L.) and barley (*Hordeum vulgare*) for centuries but re‐emerged as a potent threat in the United States in the early 1990s. This disease is caused by several fungal species, the most common being *Fusarium graminearum* (O'Donnell et al., 2000). As reported by Nganje et al. (2004), FHB caused an estimated $2.5 billion worth of losses for wheat and barley across nine US states from 1993 to 2001, primarily due to yield reduction and the accumulation of mycotoxins, particularly deoxynivalenol (DON). Besides fungicide, genetic resistance is a major lever to control the disease (M. Buerstmayr et al., 2020; Gaire et al., 2022; He et al., 2013).

The search for resistant cultivars started with the characterization of the infection types and the identification of resistant cultivars, which originated mainly from China (He et al., 2014). This paved the way for the identification of quantitative trait loci (QTLs) that are statistically associated with disease resistance and introgression of these QTLs into adapted germplasm. More than 556 QTLs have been identified for different types of FHB resistance (Venske et al., 2019), including the large effect QTLs *Fhb1* (Liu et al., 2008; Waldron et al., 1999), *Fhb5* (H. Buerstmayr et al., 2003; Xue et al., 2011), and *Fhb7* (Guo et al., 2015; Wang et al., 2020). However, a marker‐assisted selection approach can practically incorporate only a few QTLs and does not alone reach a satisfactory level of disease resistance (Arruda et al., 2016). Therefore, additional, minor genetic resistance loci need to be employed. Genomic prediction (GP) or genomic selection (Bernardo, 1994; Meuwissen et al., 2001) is a statistical method that has proven useful in enhancing genetic gain for traits with a complex genetic architecture (Heffner et al., 2009). GP consists of associating genotypic variation and phenotypic variation to predict the breeding value of new genotypes using only genotypic data. GP has become possible with the ever‐increasing availability of cheap genotyping, mainly single nucleotide polymorphism (SNP) molecular markers. GP has already been used successfully to improve FHB resistance in wheat (Adeyemo et al., 2020, 2023; Arruda et al., 2015; J. E. Rutkoski, 2019; Verges et al., 2020), as well as in many other traits and species. The advantage of GP is that it allows selection based on only genotyping of a breeding population, thus making it possible to test more genotypes, hence increasing the genetic gain (Heffner et al., 2010). Many parameters are known to affect GP accuracy, such as the statistical model (Azodi et al., 2019; Crossa et al., 2010; Heslot et al., 2012; Riedelsheimer et al., 2012), marker density (Norman et al., 2018), training set size and composition (Alemu et al., 2024; Edwards et al., 2019; Norman et al., 2018), heritability of the trait (Falconer et al., 1996), genetic relationship between training and validation set (Akdemir & Isidro‐Sánchez, 2019; Habier et al., 2010; Rincent et al., 2012; Schopp et al., 2017), and the genetic architecture of the trait (Daetwyler et al., 2010). However, the relative importance of these parameters depends on the population and trait parameters (Alemu et al., 2024; Desta & Ortiz, 2014).

After the devastating FHB outbreaks in Minnesota and the Dakotas in the early 1990s, hard red spring wheat breeding programs started to intensively breed for increased FHB resistance. Our study was focused on several public breeding programs entering lines in the Uniform Regional Scab Nursery (URSN), namely the University of Minnesota (UMN), North Dakota State University (NDSU), South Dakota State University (SDSU), Montana State University (MSU), and Agriculture and Agri‐Food Canada. Since 1995, the USDA‐ARS has coordinated the URSN, which conducts inoculated trials every year to screen for FHB resistance. Each year, the participants enter three to seven genotypes for testing in a multi‐location trial that encompasses Minnesota, North Dakota, South Dakota, and Manitoba. Even though the same wheat market class is being developed, the breeding objectives are slightly different among the breeding programs because the target population environment varies. For example, MSU has a drier environment and FHB is less of a concern there. The Uniform Regional Nursery (URN) is a much older coordinated program (began in 1929) with the same collaborators as the URSN. The main objective of the URN is to compare the best cultivar release candidates from the different breeding programs. The trials here are distributed in more locations; we used data from 1992 to 2023, and phenotyping is focused on yield and grain quality.

Other types of historical wheat scab nurseries have been studied in the context of QTL detection or GP (Boyles et al., 2023; Gaire et al., 2022; Verges et al., 2020; Winn et al., 2022, 2023). However, the URSN population, which focuses on the hard red spring wheat class in the Northern region of the United States, has never been studied so far. On the other hand, the URN population has been used in a few studies encompassing multiple historical yield trials (Morgounov et al., 2018; Zhang et al., 2022). A subset of lines tested in the URSN were genotyped, allowing us to test the efficacy of GP in these materials. The use of a diverse population such as the URSN might be challenging for GP, especially when predicting less related genotypes from breeding programs. Nevertheless, our objective was to make the most of the URSN data to enhance the predictive ability in each breeding program. For this goal, all the available phenotypic and genotypic data were compiled, consisting of eight traits related mainly to FHB, but also phenology and production. The optimum GP parameters were determined by comparing statistical methods and two genotypic arrays with different marker densities. Then, the genetic distance between training and validation sets was increased by predicting less related populations in a realistic breeding setting. To further improve the prediction with an increased genetic distance, we used a least absolute shrinkage selection operator (LASSO) algorithm to optimize the training set composition with the validation set, using a newly developed sparse selection index (SSI) (Lopez‐Cruz & de los Campos, 2021). Compared to previous studies on GP for FHB traits, the analysis was broadened to several breeding programs across the Great Plains, encompassing more genetic diversity in the hard red spring wheat market class. We harnessed the URSN regional nursery to test its ability to predict accurately FHB resistance with GP and in current breeding programs.

**Core Ideas**

- Genomic predictive ability ranged from 0.49 to 0.72 for predicting Fusarium head blight (FHB) and agronomical traits in wheat.
- Training set size had more impact on accuracy than marker density, which could be reduced to between 500 and 1000 markers.
- Training set optimization with a sparse selection index increased the accuracy of genomic prediction for two traits.
- Adding an unrelated population in the training set allows a reduction in phenotyping effort.

## 2 MATERIALS AND METHODS

### 2.1 Plant material

The present study used advanced hard red spring wheat lines developed by wheat breeding programs from URSN collaborators and phenotyped for FHB resistance. The primary focus was on the breeding lines evaluated in the historical URSN trials. However, related lines were included to test the ability of GP models to accurately predict breeding values in independent populations.

#### 2.1.1 URSN population

The URSN population comprised 222 unique lines, mainly from public university breeding programs. Each year, around 30 lines were tested in four to five locations with two common checks across all years (from 1995 to 2023). On average, lines were tested for 1.75 years, with most lines tested during a single year (201 out of 222), the check WHEATON was used in all 29 years. A total of 145 environments (a location within a year) were tested over all traits. The field design was a complete randomized block design with three blocks. The locations encompassed Minnesota, North Dakota, South Dakota, and Manitoba (Canada) for 4 and 5 years for Morden and Glenlea locations, respectively. At the end of each growing season, phenotypic data were averaged by genotype within each location and spatially corrected if needed.

Each year, the trials were inoculated with FHB, using a misted suspension of FHB inoculum for the St. Paul location (Minnesota), or spawn‐infected maize kernels for other locations, as described by Fuentes et al. (2005).

#### 2.1.2 URN population

The URN population dataset is composed of 208 lines, tested between 1992 and 2023 in 210 environments, which encompasses the same states as the URSN trials, as well as Wyoming, Nebraska, Montana, Idaho, Washington, and Saskatchewan (Canada). The dataset was subset by the lines that had been genotyped and phenotyped for FHB‐related traits. The purpose of this nursery is for the breeders to test their best genotypes, most of which are candidates for cultivar release, in various environments. To simplify, we will refer to the URN population as a breeding program. Heading date (HD) was the most frequently recorded trait, with observations in 18 locations, whereas other traits were recorded in two to four locations over the years (Figure S1). Between two and 32 (median 12) genotypes were phenotyped in each location and year. Each genotype was tested on average for 2.26 years (119 tested for 1 year, 66 for 2 years, and 23 for more than 2 years). CHRIS and MARQUIS were the two checks consistently used over the years in that nursery.

There were between 0 and 35 lines phenotyped for the first year and genotyped each year from the URN and URSN populations (Figure S2). Half of the URN and URSN genotypes were phenotyped in the last 5 years.

#### 2.1.3 UMN breeding program population

The UMN breeding program data used in this study were composed of two cohorts of 380 and 344 genotypes, from 66 and 55 parents, respectively, and with four checks, for a total of 728 unique genotypes evaluated in a preliminary yield trial in 2022 or 2023 at two locations in Minnesota (St. Paul and Crookston). Most of the genotypes were tested for 1 year in the two locations, with one replication in each location and four replications of each check (ALSEN, ROBLIN, ROLLAG, MN00269, and WHEATON). Traits evaluated in common with the URSN population were INC, SEV, DIS, visual scabby kernel (VSK), and HD (Table 1). See the trait description below (2.2.1).

**TABLE 1:** Summary of model dimensions in across‐population scenarios.

Population	BP size	URSN size	Number of markers	Genotyping		Traits
				BP	URSN	
**UMN**	728	161	35,299	GBS	90K	INC, SEV, DIS, VSK, HD
**NDSU**	679	211	30,585	90K	90K	VSK, HD, DON
**URN**	161	222	1999	3K	3K	INC, SEV, DIS, VSK, HD, DON, SW30

*Note*: The URSN size was 161 when using the 90K array and 222 when using the 3K array, except for across‐population with the NDSU population, where some individuals from NDSU were tested and genotyped with the 90K array and were in common with the URSN population and attributed to the URSN population to increase the training set size. Abbreviations: BP, breeding population (North Dakota State University [NDSU], University of Minnesota [UMN], or Uniform Regional Nursery [URN]); DIS, disease index; DON, deoxynivalenol; GBS, genotyping‐by‐sequencing; HD, heading date; INC, disease incidence; SEV, disease severity; SW30, weight of 30 spikes; URSN, Uniform Regional Scab Nursery; VSK, visual scabby kernel.

#### 2.1.4 NDSU breeding program population

The NDSU breeding program data used in this study were composed of 679 genotypes from various origins: 511 were advanced breeding lines from several breeding cohorts, 166 were historical breeding lines, and 19 were from a USDA historical panel, which comprises old founder lines used in the Dakotas and Minnesota breeding programs. The pedigree for the advanced breeding lines comprised 306 unique crosses from 306 unique parents, with LINKERT being the most used one in 45 crosses, and the largest family contained nine full sibs. Among the 306 parents for the advanced lines, 49 were in the NDSU dataset, mostly from historical breeding lines and the USDA historical panel, and 18 were part of the URSN population. For the advanced and historical breeding lines categories, genotypes were evaluated in randomized complete blocks in one to three locations from 2016 to 2023. The USDA historical panel was phenotyped in 2020 and sometimes in the past. The number of repetitions was one to three depending on the year, some advanced breeding lines were evaluated during multiple years depending on their cohort, for a maximum of 14 times (including replication). Best linear unbiased predictors (BLUPs) were predicted by fitting a mixed model with genotype effect as random and environment effects (location and year) as fixed. Traits in common with URSN were VSK, DON, and HD (Table 1). Lines that were tested in URSN trials were removed from that population.

### 2.2 Phenotyping and data analysis

#### 2.2.1 Description of traits

For the URSN population, eight traits were included in the analysis, namely, disease incidence (INC [%]), severity (SEV [%]), disease index (DIS [%]) corresponding to incidence multiplied by severity (Adeyemo et al., 2020), VSK after harvest (VSK [%]), DON concentration (ppm), HD (in Julian day), micro‐test weight (mTWT [g]), corresponding to the weight of seeds contained in a copper vessel of 15.7 mL, and weight of 30 spikes (SW30 [g]). A summary of traits is available in Table S1. Phenotypic structure among subpopulations was assessed by a principal component analysis (PCA), projected on the first two axes.

#### 2.2.2 Mixed model analysis

The following mixed model was applied for each trait and within each population:

(1) $$y_{ijk}=1\mu +\underline{g_{i}}+l_{j}+y_{k}+{\left(l:y\right)}_{jk}+\varepsilon_{ijk}$$

Here, $y_{ijk}$ is the phenotypic value in an entry‐mean basis for the genotype i, the location j, and the year k. Genotypic effect $\underline{g_{i}}$ was considered as random, while other effects were fixed. Namely, $l_{j}$, *y_k_*, and ${\left(l:y\right)}_{jk}$ are the fixed effects of location (up to 11 levels for the URSN population depending on the trait), year (29 levels), and year‐by‐location interaction (145 levels). $\underline{g_{i}}$ and $\varepsilon_{ijk\;}$ were assumed independent, identically distributed, and followed a normal distribution, with $\sigma_{u}^{2}$ and $\sigma_{e}^{2}$ as variance parameters, respectively. The selection of significant effects was done using the lmerTest R package (Kuznetsova et al., 2017). Random effects were selected based on a likelihood ratio test, and fixed effects were based on the Fisher test. The reliability was estimated as: $i^{2}=\frac{\sigma_{u}^{2}}{\sigma_{e}^{2}/n}$, where n is the harmonic number of genotype replication (Holland et al., 2003). When multiple populations were analyzed together, BLUPs of phenotypic values were centered and scaled in each population before merging.

### 2.3 Genotyping

#### 2.3.1 For URN and URSN populations

A sample of individuals from the URN and URSN populations was genotyped using two genotypic arrays, with 90,000 and 3000 markers (referred to as the 90K array and the 3K array, respectively). After genotyping, GenomeStudio software was used to determine SNP alleles after manual curation to ensure that polymorphic sites were correctly called. The markers were assigned positions based on Basic Local Alignment Search Tool on the IWGSC RefSeq v2.1 reference genome (Zhu et al., 2021).

For the 90K array, 230 individuals were genotyped, with an initial number of markers of 81,587 after pre‐processing. Duplicated and non‐informative markers were removed, along with genotypes or markers with more than 50% missing data. A minor allele frequency threshold of 5% was applied and only the genotypes that were phenotyped were retained, resulting in 39,268 SNPs for 230 genotypes (among them, 161 have been tested in the URSN and 89 in the URN trials). We applied the same workflow for the 3K genotypic array, resulting in 1999 SNPs for 390 genotypes (among them, 222 have been tested in the URSN and 216 in the URN trials). Missing data were imputed within each genotypic array (both for the URN and URSN populations) with Beagle v5.4, without reference (Browning et al., 2021). PCA projected on the first two axes was used to investigate the genetic structure. (Table 1).

There were 119 markers in common between the two genotypic arrays after marker filtering. To test the effect of genotypic array and marker density, a subset of SNPs was sampled from the 39,268 of the 90K array and repeated 10 times. Several densities were tested: 100, 500, 1000, 2000, and 10,000. Besides, 161 genotypes out of 222 were subsampled from the 3K array to test the effect of the number of genotypes.

#### 2.3.2 For breeding program populations

##### UMN breeding program

Lines were genotyped at F5 or F6 stages, and then lines were selected based on GP for FHB susceptibility and other traits. Genotyping information was acquired with genotyping‐by‐sequencing (GBS) (Elshire et al., 2011) for the 728 genotypes. After merging the genotyping data across multiple years' files, 4106 SNPs remained after sub‐setting for the 728 lines studied here. More details about the GBS method are available in Supporting Information Methods S1. Genotyping data from GBS and the 90K array (before imputation) were merged with TASSEL 5 (Bradbury et al., 2007). Beagle v5.4 was used for imputing the numerous missing data with default parameters, based on the physical position of the SNPs in the genome (Browning et al., 2021, 2018). After imputation and filtering, the genomic matrix comprised 728 genotypes for 35,299 SNPs (Table 1).

The quality of imputation was assessed within the URSN population, between the two genotypic arrays (Supporting Information Methods S2). Since the 3K genotypic array has approximately the same number of markers as the UMN GBS genotyping, it provides an estimate of the expected imputation accuracy. The accuracy was estimated as the ratio between the sum of the true positives and true negatives and the sum of the true and false positives and true and false negatives. The mean accuracy of imputation across all markers was 0.84 (median = 0.94), with averaged accuracy per chromosome ranging from 0.832 to 0.87 (Figure S3).

##### NDSU breeding program

The genotyping was conducted using the 90K genotyping array, with 81,587 SNPs left after filtering for 679 genotypes. Genotypic data were merged with the initial URSN variant calling format file (before imputation), and imputation was performed using Beagle v5.4 (Browning et al., 2021). Finally, a filter cutoff threshold of 5% minor allele frequency was applied, resulting in 30,585 SNPs for 679 genotypes (Table 1). Among those, some of them have been tested in URSN trials, we attributed them to the URSN population, resulting in 211 individuals in the URSN population for that specific case (Table 1).

### 2.4 Genomic prediction

#### 2.4.1 Description of GP methods

Eight statistical methods were compared for GP within the URSN population. The R statistical software version 4.4 was used to perform all the analyses (R Core Team, 2024).

- Ridge regression BLUP (RR‐BLUP) (Endelman, 2011; Meuwissen et al., 2001). The BLUP model is as follows:
(2) y=Xβ+Zu+e
  with y as the vector of genotypic values estimated from Equation (1), β as the vector of fixed effects (identity matrix here), u as the random effects of markers, assumed $u∼N(0,\sigma_{u}^{2})$, with X as the marker matrix, coded in −1, 0, 1, with genotypes in rows and markers in columns. RR‐BLUP consists of a penalized linear regression, with a shrinkage parameter $\lambda =\sigma_{e}/\sigma_{u}$.
- Reproducing kernel Hilbert space (RKHS) is a semi‐parametric statistical method that models nonlinear effects (de los Campos et al., 2010; Gianola et al., 2006). It relies on a Gaussian kernel, computed as a spatial distance between genotypes, such as $K_{i,j}=exp\left(-hd_{ij}^{2}\right)$, with h a bandwidth parameter fixed to 1 here, and $d_{i,j}$ the Euclidean distance between genotypes i and j based on molecular markers. The RKHS model was fitted using the BGLR R package, with 6000 iterations and a burn‐in phase of 1000. An extension of RKHS was also tested: RKHS kernel averaging (RKHS‐KA), with varying bandwidth parameters (0.01, 0.1, 0.4, 0.8, 1.5, 3, and 5). In RKHS‐KA the six kernels are “averaged” to form a new kernel (de los Campos et al., 2010; Morota & Gianola, 2014).
- Bayes A and Bayes B are two Bayesian methods implemented with the BGLR R package (Pérez & de los Campos, 2014). They rely on different distribution assumptions for the regression coefficient (i.e., marker effect) estimation. Bayes A and Bayes B both use a scaled inverted chi‐square distribution for genetic variance prior. The difference between Bayes A and B is that in B, there is a proportion π of markers that are assumed to have a 0 variance (Meuwissen et al., 2001).
- LASSO (Tibshirani, 1996), is a penalized linear regression method, as RR‐BLUP. Compared to the ordinary least square regression, it adds a shrinkage with an L1 norm, such that it minimizes the penalized sum of squares: $\sum_{i=1}^{n}{\left(y_{i}-\;\sum_{j=1}^{p}z_{ij}u_{j}\right)}^{2}+\lambda \sum_{j=1}^{p}\left|u_{j}\right|$. The shrinkage parameter λ was estimated by an inner cross‐validation, using the glmnet R package (Friedman et al., 2023). It performs variable selection; hence this method is more adapted to a simple genetic architecture (i.e., with a small number of QTLs).
- Random forest (RF) is a popular machine‐learning regression and classification method (Breiman, 2001). It performs classification on trees built on bootstrap sampling of data and aggregates the results of all trees. Hence, it is less likely to overfit and yield results with small variance. The R package caret (Kuhn, 2008) was used to tune the hyperparameters and randomForest R package (Liaw & Wiener, 2002) to fit the model. The number of trees was set to 100 and the number of random variables selected at each tree was tuned, with a grid of 10 values between one and one‐third of the total number of markers.

GP was run with a 10‐fold cross‐validation repeated 10 times. Predicted values were aggregated over all folds (*n* = 222), and predictive ability was calculated as the Pearson correlation between predicted and observed phenotypic (BLUP) values. The predictive ability was calculated across all folds, thus having 10 values for each trait and prediction method. To study the impact of the genetic structure within the URSN population, we selected in turn each subpopulation from the URSN population to be part of the validation set. Hence, each training set was reduced by the size of each subpopulation studied (UMN, NDSU, SDSU, and MSU with sizes of 67, 43, 66, and 37).

#### 2.4.2 Training set optimization

For training set optimization, the new SSI was used (Lopez‐Cruz & de los Campos, 2021), implemented by the R package SFSI (Lopez‐Cruz et al., 2020). For each genotype in the validation set, it searches for the best set of genotypes to include in the training set, using a similar algorithm as the LASSO. The hyperparameter λ was optimized by inner cross‐validation by maximizing the predictive ability while having a minimum of 60 genotypes selected. This parameter determines the sparsity of selection, that is, how many lines will be selected from the training set. Three metrics were averaged over all validation set genotypes: (i) the training set size, (ii) the predictive ability, and (iii) the percentage of lines originating from the URSN population (when multiple populations were available in the training set). Since the optimized training set is specific to each individual in the validation set, this method's purpose is not to reduce the overall training set size but rather to use the best set of lines from a given training set.

#### 2.4.3 Scenarios tested for breeding programs

**FIGURE 1:**
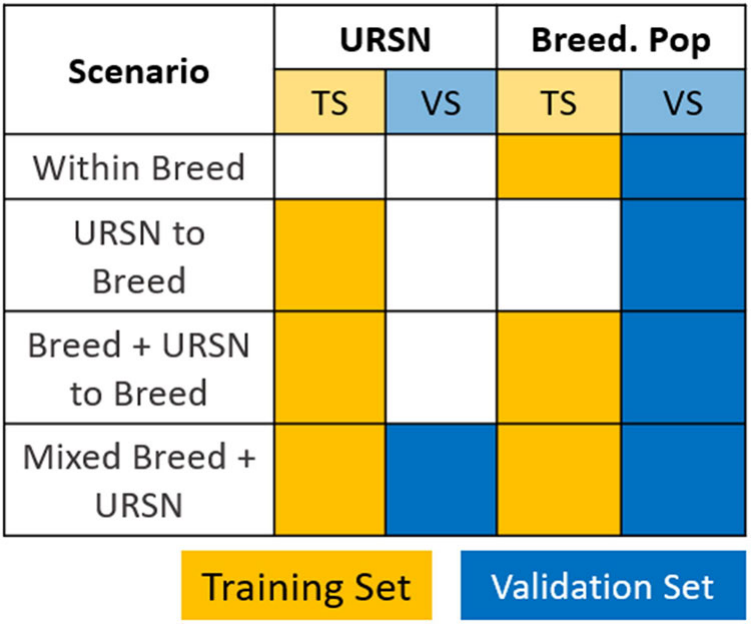
Description of tested scenarios. Breed. Pop, breeding population; TS, training set; URSN, Uniform Regional Scab Nursery; VS, validation set.

For assessing the potential of the URSN population to be useful in predictions for the breeding programs, several scenarios were compared (Figure 1):

– Within Breed: It includes random cross‐validation (10‐fold repeated 10 times) within each breeding population (NDSU, UMN, and URN).
– URSN to Breed: It trains the GP model in URSN and predict the breeding population (without cross‐validation).
– Breed + URSN to Breed: It trains the GP model using both URSN and breeding population data and predict only breeding population genotypes. 10‐fold cross‐validation repeated 10 times was used to sample genotypes in the validation set.
– Mixed Breed + URSN: It uses all available data to train and predict with random cross‐validation (10‐fold repeated 10 times).

Cross‐validation was repeated 10 times when applied. RR‐BLUP and RKHS were used as GP methods for those scenarios. Models were fitted separately for each breeding program. The effect of the training set size was tested by using a subset of UMN and NDSU breeding populations as training sets in Within Breed and Breed + URSN to Breed scenarios. The sample sizes were 25, 50, 100, 200, 300, 400, and 500. The whole URSN population was added to the training set for the Breed + URSN to Breed and in both scenarios, only breeding population genotypes were in the validation set.

Different comparisons were performed using the described dataset. First, the predictive ability of the GP methods within the URSN population was compared for both genotypic arrays. Second, the effect of marker density by decreasing the number of markers was tested. Finally, the usefulness of the URSN population to enhance breeding program efficiency was tested with the efficiency of training set optimization.

## 3 RESULTS

### 3.1 Populations analysis

#### 3.1.1 URSN population

**FIGURE 2:**
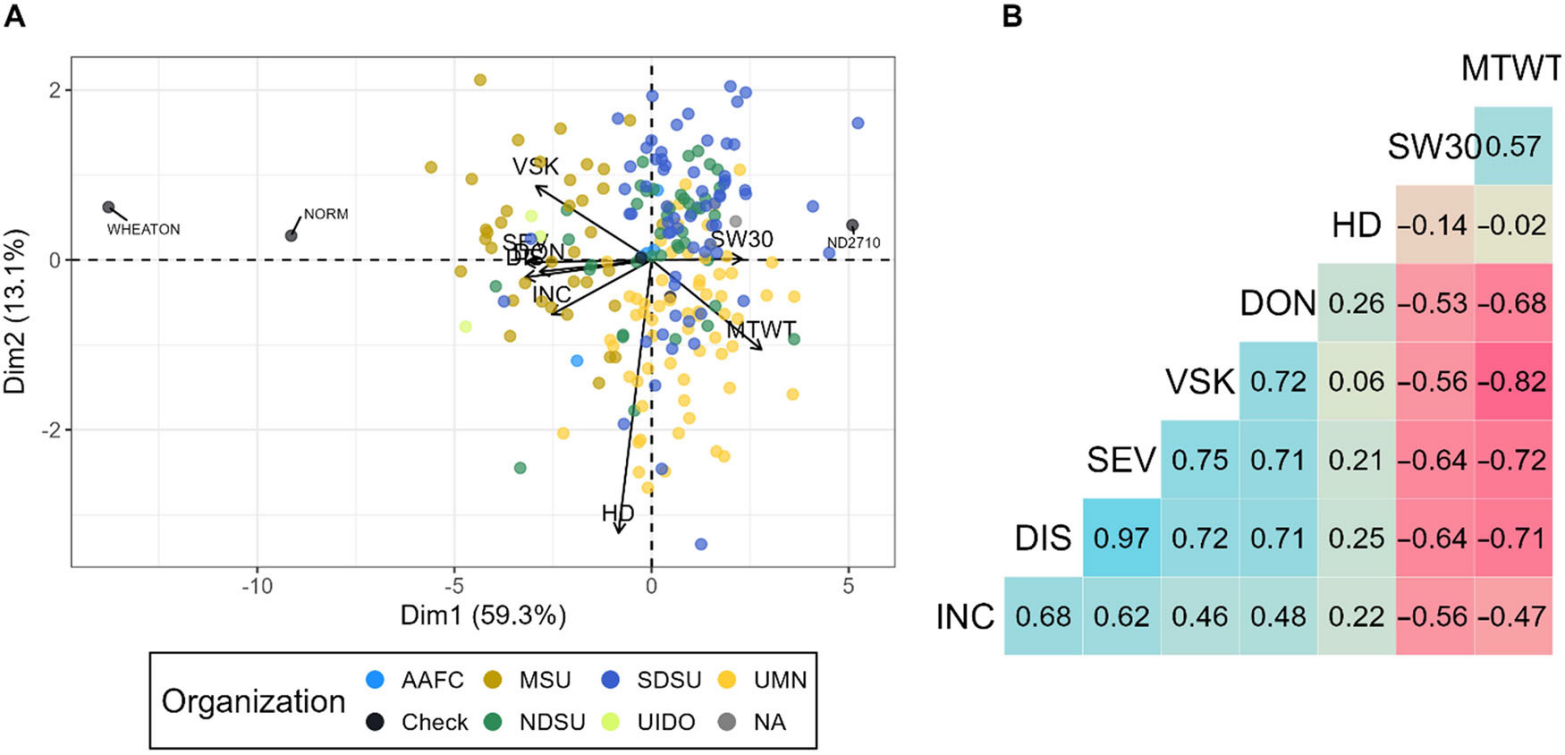
Analysis of best linear unbiased predictors (BLUPs) from phenotypic data. (A) Principal component analysis (PCA) biplot of the phenotypic structure, labeled genotypes are checks. (B) Genetic correlations among traits. DIS, disease index; DON, deoxynivalenol; HD, heading date; INC, incidence; MTWT, micro‐test weight; SEV, severity; SW30, weight of 30 spikes; VSK, visual scabby kernel.

There was a mild phenotypic structure among the organizations participating in the URSN (Figure 2A). FHB‐related traits were loaded on the first axis, with higher FHB disease for MSU and private companies’ genotypes on the left side of the PCA in Figure 2A. The second PCA axis was more explained by HD and production traits (mTWT and SW30). There were strong positive and significant correlations ranging between 0.46 and 0.97 among FHB traits, weaker correlations between HD and other traits, and strong negative correlations between other agronomic and FHB traits. The reliability values were moderate to high and ranged between 0.438 for INC and 0.877 for HD (Table S1).

We then studied the genetic structure among the entries and found the first two axes of the PCA explained 38.7% of the variance (Figure S4). We observed a higher genetic relatedness within organizations (Figures S3 and S4) and genetic differentiation among organizations, particularly for NDSU and MSU (Figure S5). Furthermore, the URN and URSN nurseries were completely overlapping (Figure S5).

#### 3.1.2 Other breeding programs

The NDSU breeding program (NDSU_BP) population showed some genetic relatedness with a subset of URSN genotypes (Figure S6). The UMN breeding program (UMN_BP) was structured in two subpopulations corresponding to preliminary yield trial cohort from two different years. The URSN population showed more genetic relatedness with the NDSU_BP than with the UMN_BP population (Figure S6).

### 3.2 Comparison of GP methods within the URSN population

Seven GP models were compared using cross‐validation within the URSN population using the 3K genotypic array on 222 genotypes (Figure S7). The results showed an accuracy above 0.49 for all traits and up to 0.72 for DIS and SEV with RKHS. DIS was the best‐predicted trait on average (mean accuracy of 0.702), and INC was the worst‐predicted trait (mean accuracy of 0.475). There was no correlation (*r* = 0.075) between the trait reliability and the average predictive ability. The seven methods performed rather similarly. RKHS was the best method, with the best accuracy for four out of eight traits and an average accuracy of 0.635. rrBLUP was ranked fourth, after RKHS, RKHS‐KA, and Bayes A. The comparison of predictive ability (Figure S7) between rrBLUP and RKHS showed that the values were highly correlated, with a small advantage for RKHS for the HD and DON traits. Random forest and LASSO were the two worst‐performing methods, with average predictive abilities of 0.621 and 0.526, respectively (Figure S8). For the following results, our analysis will focus on the RKHS method and RR‐BLUP method for the training set optimization part.

**FIGURE 3:**
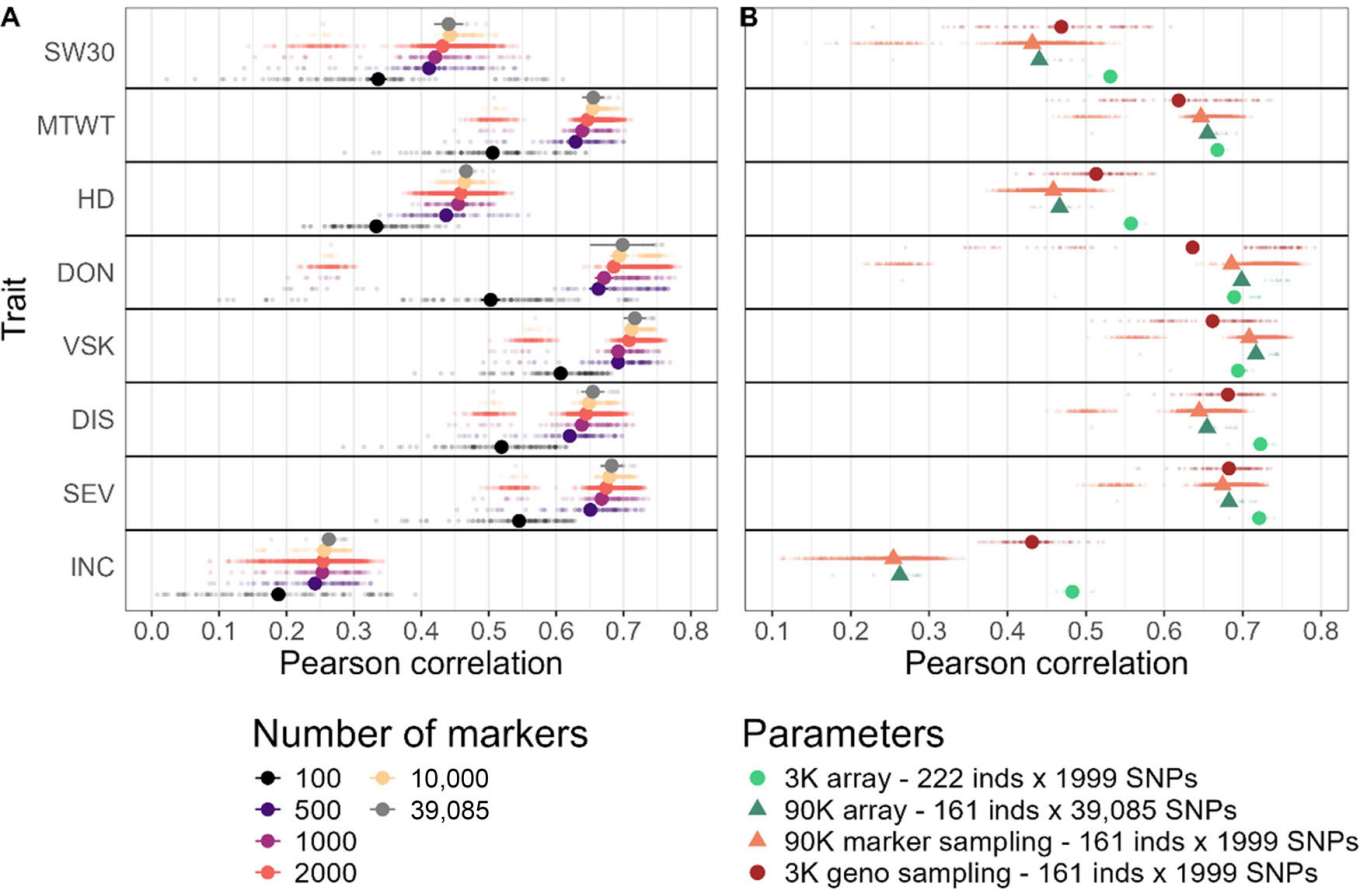
Comparison of predictive ability for different genotypic arrays and marker density for reproducing kernel Hilbert space (RKHS) genomic prediction method with 10‐fold cross‐validation. (A) Sampling of markers from the 90K genotypic array, which comprises 39,085 single nucleotide polymorphisms (SNPs) and 161 individuals. (B) Comparison between genotypic arrays (3K with circle and 90K with triangle) with initial datasets (in light and dark green), using a subsample of markers to match the 3K density (light orange), and using a subsample of individuals to match the number of individuals in the 90K array (dark orange). DIS, disease index; DON, deoxynivalenol; HD, heading date; INC, incidence; inds, individual (lines); MTWT, micro‐test weight; SEV, severity; SW30, weight of 30 spikes; VSK, visual scabby kernel.

The ranking of methods was similar when using the 90K genotypic array (Figure S9). The predictive ability of the traits was similar to those obtained with the 3K array, except for INC and HD for which predictive ability was lower using the 90K compared to the 3K (Figure 3B). INC was the least predicted for both genotypic arrays (Figures S8 and S9). The observed versus predicted genotypic values were plotted to assess the effect of the structure between organizations (Figure S10). Indeed, genomic estimated breeding values from MSU genotypes were predicted to have higher disease and were more distinct from other organizations.

### 3.3 Impact of genotypic array and marker density

The impact of marker density on predictive ability within the URSN population was investigated using the 90K genotypic array, and beyond a threshold, there was a drop in predictive ability (Figure 3A). That threshold depended on the trait and was between 100 and 500 markers. Between 1000 and 10,000 markers, there was a small, steady increase in accuracy until the total set of 39,085 markers.

The impact of the genotypic array with marker density was studied, comparing the predictive ability of the two initial genotypic arrays (3K with 222 genotypes and 90K with 161 genotypes), with the prediction from a random sampling of 1999 markers from the 90K array, and from a random sampling of 161 individuals from the 3K array. The results showed that, on average, the 90K genotypic array had comparable predictive ability as a decreased density of 1999 markers (Figure 3B). The sampling of 161 individuals from the 3K array led to a decrease in predictive ability compared to 222 individuals from the same array. The subset of markers and the subset of individuals, both datasets having 161 individuals and 1999 SNPs from the 90K and the 3K array, respectively, showed significant variations. For INC, HD, and SW30, the predictive ability was higher for the dataset from the 3K array, whereas for DON and VSK, there was a decrease in predictive ability compared to the 90K array. This suggests an effect of i, the individuals included in each array, as well as the choice of markers in the 3K array.

### 3.4 Predicting among organizations in URSN

**FIGURE 4:**
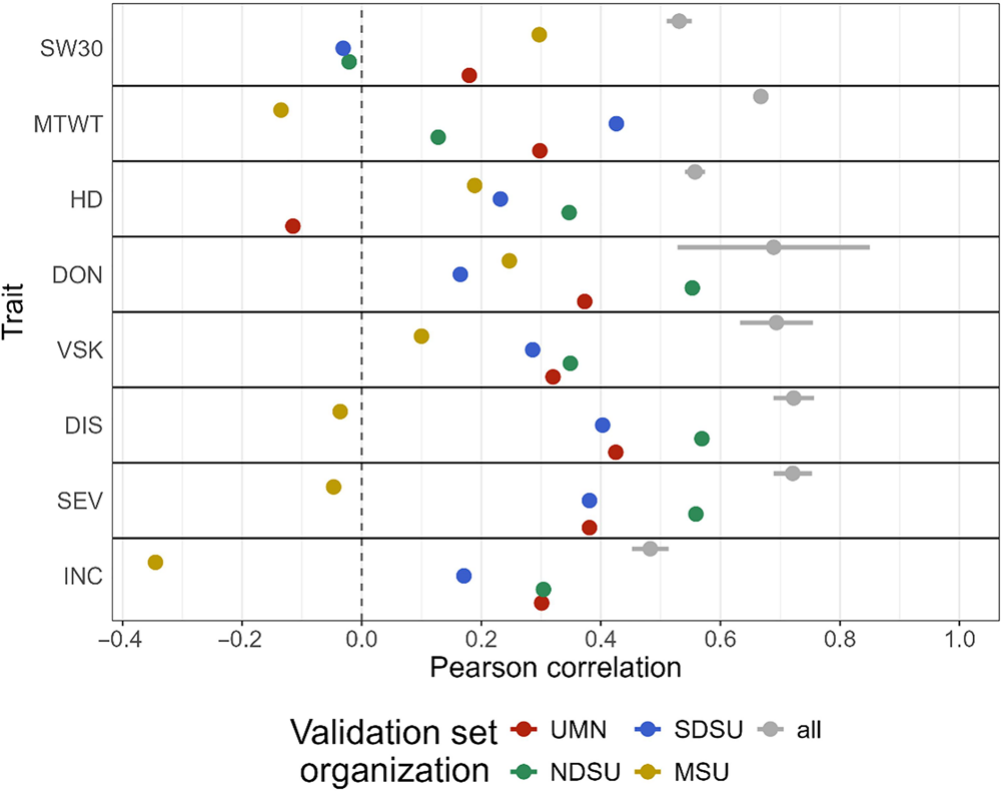
Genomic prediction using reproducing kernel Hilbert space (RKHS) within the Uniform Regional Scab Nursery (URSN) population. Each organization was in turn absent from the training set and predicted. The reference random cross‐validation predictive ability was displayed in gray, with standard deviation around the mean. RKHS method and 3K genotypic array were used. MSU, Montana State University; NDSU, North Dakota State University; SDSU, South Dakota State University; UMN, University of Minnesota.

The ability of the URSN population to predict FHB traits in a specific organization (UMN, NDSU, SDSU, or MSU) was investigated by excluding lines from a specific organization from the training set and including only those genotypes in the validation set (Figure 4). Compared to the reference random cross‐validation, there was a decrease in accuracy for all traits (Figure 4). However, the magnitude of the decrease depended on the population and the trait to be predicted. NDSU had the highest predictive ability (mean of 0.38, *n* = 43), then UMN (mean of 0.29, *n* = 67), SDSU (mean of 0.26, *n* = 66), and lastly MSU (mean of 0.03, *n* = 37). These results were consistent with the genetic relatedness among organizations, with less relationship between MSU and the other organizations among the URSN population (Figure S4).

### 3.5 Predicting other breeding programs

The usefulness of the URSN population was tested with several prediction scenarios (see Section 2.4.3). First, the RKHS and RR‐BLUP GP methods were compared on all scenarios, populations, and traits (Figure S11). A significantly (*p*‐value < 2.2 e^−16^) higher predictive ability was observed for RKHS compared to RR‐BLUP, with a difference of 0.03. The results hereafter will be restricted to the RKHS method.

**FIGURE 5:**
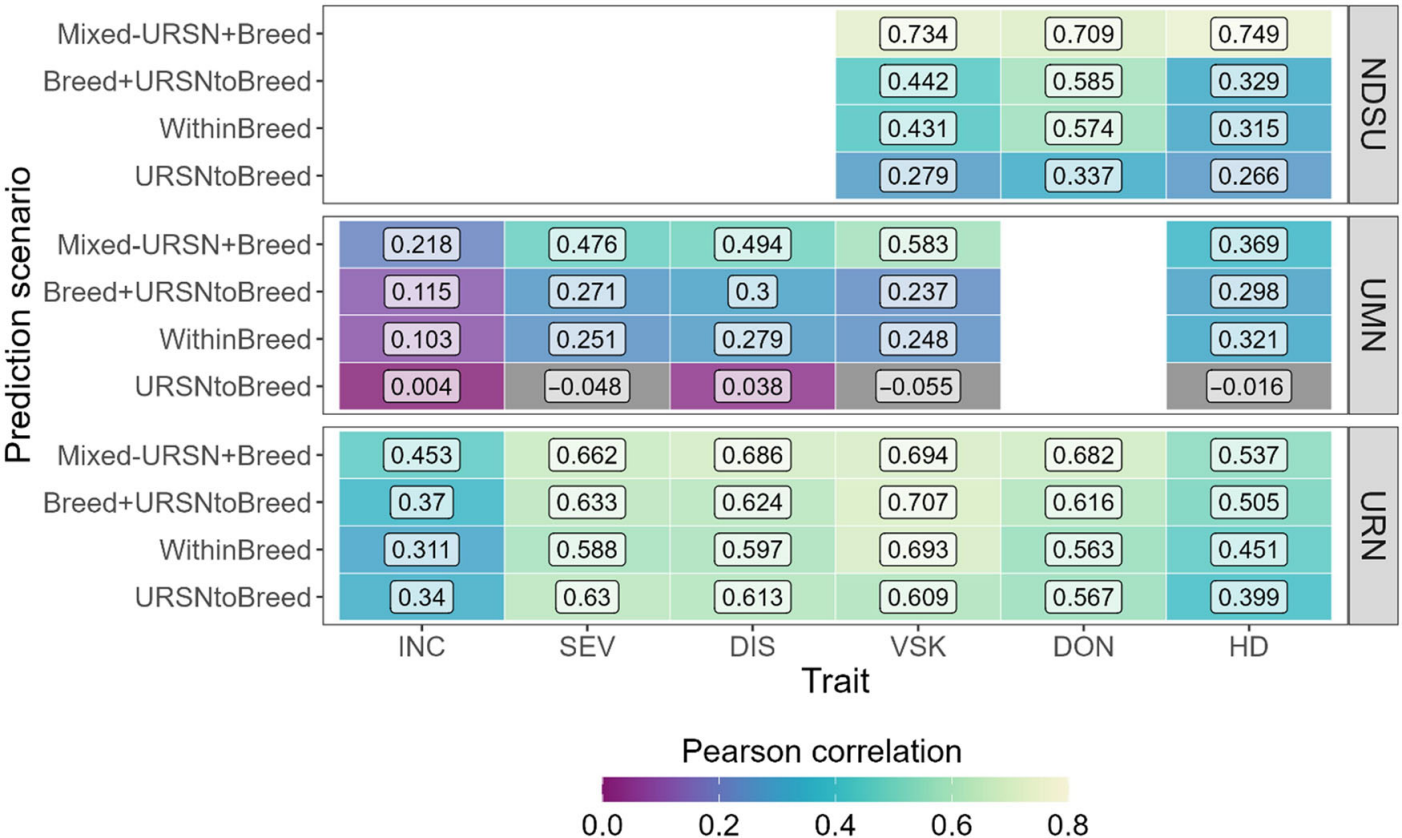
Genomic prediction in three breeding programs (North Dakota State University [NDSU], University of Minnesota [UMN], and Uniform Regional Nursery [URN]), with or without the Uniform Regional Scab Nursery (URSN) population as part of the training set. Each cell was labeled with the mean predictive ability for each scenario and trait, using reproducing kernel Hilbert space (RKHS) genomic prediction (GP) method. Blank sections correspond to traits that were not phenotyped in breeding program populations. DIS, disease index; DON, deoxynivalenol; HD, heading date; INC, incidence; SEV, severity; VSK, visual scabby kernel.

Overall, predictive ability was highest for the URN population and lowest for the UMN breeding program (Figure 5). The URN population consistently showed stable predictive ability across all scenarios, while the UMN and NDSU populations experienced significant variation. In the Within Breed scenario, used as a reference for running GP in cross‐validation, predictive ability values ranged from 0.103 for INC and UMN to 0.694 for VSK and URN. For UMN and NDSU populations, there was an average decrease in predictive ability of 0.25 and 0.15, respectively, between the Within Breed and URSN to Breed scenarios. The URSN to Breed scenario, where URSN was the training set and breeding population the validation set, was the most challenging, leading to the lowest predictive ability, particularly in the UMN population, where values were close to zero for all traits (Figure 5).

The Breed + URSN to Breed scenario (i.e., adding URSN to the training set) resulted in predictive ability comparable to the Within Breed scenario, except in the URN population, where the mean predictive ability increased from 0.53 to 0.58. The highest mean accuracy for all populations was observed in the Mixed URSN + Breed scenario, especially in the NDSU population, where predictive ability reached 0.749 for HD. This was expected due to the larger training set and the strong genetic and phenotypic structure in the combined populations, which drove up the predictive ability.

**FIGURE 6:**
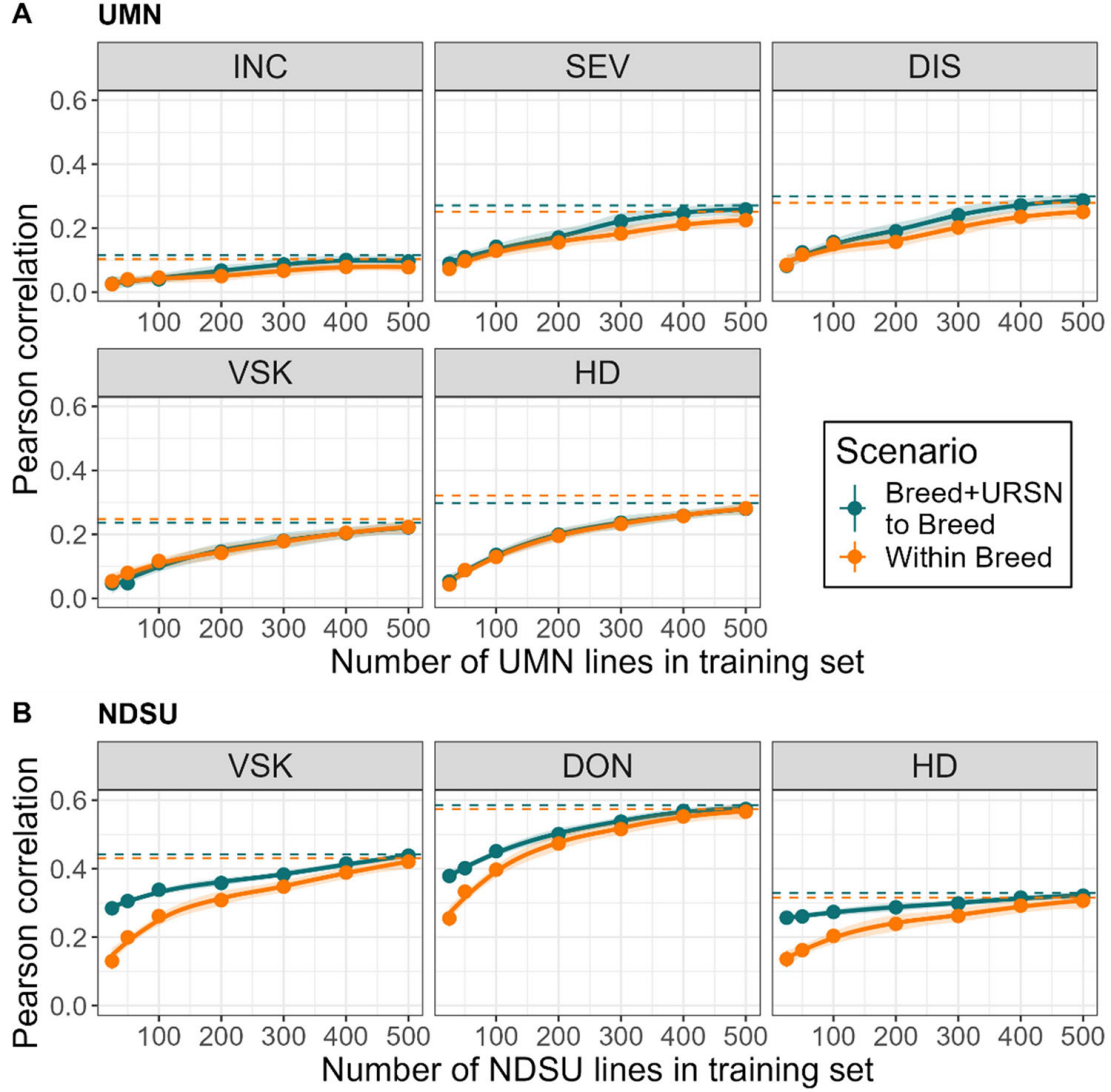
The predictive ability for University of Minnesota (UMN) (A) and North Dakota State University (NDSU) (B) populations with the reproducing kernel Hilbert space (RKHS) method, using a mix of the breeding population and Uniform Regional Scab Nursery (URSN) genotypes in the training set, with varying numbers of breeding lines. The dashed lines are the predictive ability using the full training set (random cross‐validation), for each scenario. The smooth line is a loess regression over training set size, and the shading is the 95% confidence interval around this regression. DIS, disease index; DON, deoxynivalenol; HD, heading date; INC, incidence; SEV, severity; VSK, visual scabby kernel.

To further dissect the potential of the URSN population to improve prediction accuracy, the number of genotypes from the breeding population was progressively increased in the training set for the Within Breed and Breed + URSN to Breed scenarios (Figure 6). Our hypothesis was that the URSN population might be useful to decrease the phenotyping effort in the breeding program. To test that, the same number of breeding program genotypes were sampled in both scenarios and added URSN in the training set for the second scenario (Figure 6). The results showed that predictive ability indeed increased with increased training set size for all traits and populations and for both scenarios. The difference between the two scenarios varied depending on the trait and the population.

The superiority of the Breed + URSN to Breed scenario was more pronounced for the NDSU population (Figure 6B). In that case, the Breed + URSN to Breed performed better than Within Breed with a training set size from the NDSU breeding program between 50 and 500, depending on the trait. The largest difference of predictive ability was for the NDSU population and the smallest training set size of 25 with an average difference of 0.14 between the two scenarios over the traits (with a training set of 25 NDSU lines compared to 25 NDSU lines added to the URSN population). For the UMN population, most of the difference between the scenarios was observed for training set sizes of 200 and 300. Therefore, adding URSN to the training set increased the predictive ability when the training set size was reduced, dependent on the trait and method.

### 3.6 Training set size and optimization

**FIGURE 7:**
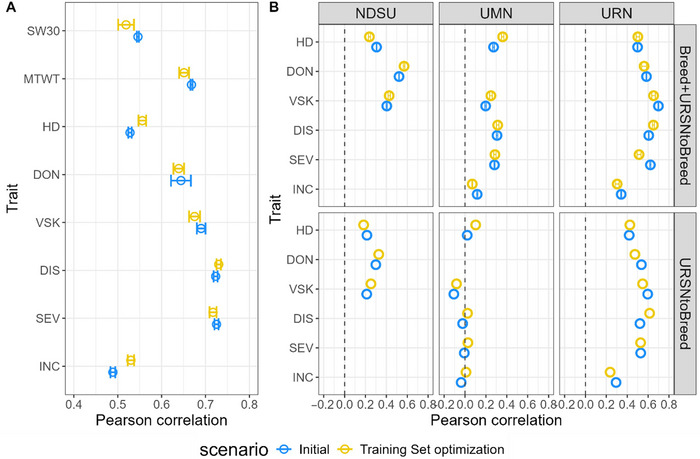
Effect of training set optimization using a sparse selection index (in yellow) or no optimization (in blue) with ridge regression BLUP (RR‐BLUP) with different prediction scenarios. (A) Within Uniform Regional Scab Nursery (URSN) cross‐validation. (B) Prediction in North Dakota State University (NDSU), University of Minnesota (UMN), and Uniform Regional Nursery (URN) breeding program with two different training sets for each: URSN or URSN and breeding program. Error bars mean predictive ability ± standard error. BLUP, best linear unbiased predictor; DIS, disease index; DON, deoxynivalenol; HD, heading date; INC, incidence; MTWT, micro‐test weight; SEV, severity; SW30, weight of 30 spikes; VSK, visual scabby kernel.

Since the populations studied here display some genetic structure (Figure S4), a training set optimization algorithm was applied. Within the URSN population (random cross‐validation) (Figure 7A), there were minor or no improvements in predictive ability when using training set optimization. The average predictive ability increased by 1.8 percentage points. Most of the traits showed no significant improvement, except significant improvement for INC (*p*‐value of *t*‐test = 9.6e‐5, difference of 0.041) and HD (*p*‐value = 0.014, difference of 0.028). For both of these traits, the average optimized training set size was composed of 62 genotypes.

Furthermore, training set optimization was applied to across‐population settings in Within Breed and Breed + URSN to Breed (Figure 7B). The aim was to determine if a subset of URSN or breeding program lines could achieve higher predictive ability with training set optimization. There was no overall difference between the populations when comparing training set‐optimized prediction versus regular prediction (Figure 7B).

Training set sizes after optimization varied a lot across traits, scenarios, and populations (Figure S10), partly due to availability of data among the populations. The optimized training set sizes were lower in the URN population (between 52 and 154) and were similar between scenarios and traits for that population, except for HD trait and Breed + URSN to Breed and Mixed URSN + Breed scenarios with around 150 genotypes selected. For the two latter scenarios, more than half of the selected genotypes were coming from the URSN population, roughly corresponding to the ratio of URN and URSN genotypes (Figure S12). The optimized training set sizes were much higher in the UMN population, with values up to around 800 for SEV and DIS and when both URSN and UMN were available as training sets. However, training set sizes varied a lot across traits and scenarios. For example, the optimized training set size was between 61 and 115 across scenarios for HD and the UMN population. For the NDSU population, the maximum training set size was 316 for HD and the Within Breed scenario. In all populations, fewer genotypes were generally selected in the training set for the URSN to Breed scenario (52–194), also because of the lower number of lines from the URSN population (*n* = 222). The proportion of URSN lines in Mixed URSN + Breed and Breed + URSN to Breed remained quite stable across traits and for the UMN and NDSU populations (10%–25%) (Figure S10).

## 4 DISCUSSION

In this study, the potential of GP in hard red spring wheat breeding programs to improve FHB resistance was evaluated, leveraging historical phenotypic data. The nonlinear method RKHS was slightly better than the other GP methods. Decreasing marker density to a threshold of 1000 SNPs did not decrease the accuracy of GP. Our main population, the URSN, was structured into sub‐populations, and separating them in GP resulted in a moderate to strong decrease in accuracy. Finally, additional populations from current breeding programs were incorporated. Adding genotypes from the URSN population improved the predictive ability when the training set size was small. However, training set optimization did not improve the predictive ability for most traits.

### 4.1 Comparison of populations

Several wheat populations were compared in this study, with contrasting composition parameters. The URN and URSN populations have been phenotyped over many years in multiple locations. Genotypes from those populations reflect the most promising lines in the breeding programs for cultivar release and FHB resistance, respectively. Hence, it led to a dataset that is representative of the spring wheat breeding programs in the US Midwest, but not necessarily adapted for GP. Furthermore, there was a small number of lines genotyped in the URSN population compared to breeding programs. Using GP with the URSN as the training set and URN as the validation set gave a good predictive ability, likely because there was a high genetic relatedness between those populations, and genotyping was performed with the same genotypic array.

The NDSU population comprised advanced lines from the North Dakota breeding program and older founder lines. Genotyping data were acquired with the same 90K genotypic array as the URSN population, resulting in common markers with the URSN population. It is not surprising then that the lines overlapped well. Compared to the URN population, there was a stronger decrease in predictive ability for the URSN to Breed scenario (Figure 5), which can be explained by a higher genetic distance between training and validation sets.

The UMN population comprised preliminary yield lines from two cohorts, phenotyped over 2 years. This structure is highly visible in Figure S5, and a larger distance with the URSN population was noticeable. This might be due to the imputation step because genotyping was done by GBS on the UMN breeding population; thus, there were a few markers in common with the genotypic array. In that context, it was not surprising that the URSN population was unsuitable for predicting breeding values for UMN (Figure 5). Within URSN population, the imputation accuracy between the two genotypic arrays was rather high (mean of 0.84, median of 0.94), despite the large number of markers to impute (Supporting Information Methods S2; Figure S3). Nevertheless, we expect a smaller imputation accuracy in the UMN breeding program population since the URSN and the UMN populations are less related. Besides, the use of Beagle for imputation might be responsible for the genetic structure we observe between the URSN and UMN populations when we impute. Thus, the adoption of a common marker platform across different breeding programs may facilitate the incorporation of data for GP (Sneller et al., 2021). The predictive ability within the UMN breeding population was low (between 0.103 and 0.321), probably because the genetic variability in this population was smaller, with genotypes already selected for low FHB susceptibility.

### 4.2 Genetic structure and distance had the greatest impact on GP predictive ability

The results presented here allowed us to test the effect of many parameters on predictive ability: genetic architecture (up to eight traits studied), GP method, training set size and composition, heritability, genetic structure, marker density, and genetic relatedness between the training and validation sets. Among all parameters compared, marker density and genetic relatedness had the greatest impact on predictive ability. Indeed, there was a quite stable predictive ability between the traits, GP methods, and genotypic arrays (Figure 3; Figures S8 and S9). The close performance of GP methods was already observed in other studies (Azodi et al., 2019; Brault et al., 2021; Resende et al., 2012; Yamamoto et al., 2017). Despite its popularity, RR‐BLUP was not the best‐performing method in our study. RKHS, a nonlinear method, was on average slightly better than rrBLUP within the URSN population for HD and DON traits (Figure S7), and when predicting other breeding programs (Figure S11). The decrease in marker density between the two genotypic arrays did not impede the predictive ability when comparing similar training set sizes (Figure 3B), suggesting that the linkage disequilibrium was strong enough to tag causal variants in the lower density, cheaper 3K array. This is expected in self‐pollinated species such as wheat and in genotypes coming from breeding programs. However, using two different genotyping techniques (arrays and GBS) decreased prediction accuracy in our study with the UMN breeding population, probably because of the lack of common markers and therefore a less accurate marker imputation.

GP accuracy was higher for FHB traits than in other studies (Adeyemo et al., 2020, 2023; Desta & Ortiz, 2014). This could be explained by the phenotypic and genetic structure within the URSN population (Figure 2; Figure S4). As shown in Figure S10, the predictive ability was driven by the phenotypic structure of the URSN subpopulations. For example, the MSU subpopulation from the URSN population displayed a higher susceptibility to FHB and is also more apart from the other URSN subpopulations (UMN, NDSU, and SDSU) (Figure S4). Then, the MSU individuals will be predicted with higher susceptibility by GP. This results in a slight overestimation of what would have been obtained within each subpopulation. Comparing the genetic relatedness among the populations studied, the UMN and NDSU breeding programs showed low relatedness with the URSN population, whereas the URN population was highly related and had the same genetic structure. Nevertheless, this population was also phenotyped during different years. This specific experimental design allowed us to hypothesize that the genetic relationship between the training and validation set was the most significant factor impacting the predictive ability. However, one should remain careful about the measure of genetic relationship since it was imprecise due to the changes in allele frequency and the use of marker imputation.

Training set size was not purposefully studied, but it is accepted that a size of around 200 genotypes with a narrow genetic diversity (such as in a breeding program) would be enough to ensure good predictive ability (Adeyemo et al., 2020). Strikingly, predictive ability reached a plateau between 400 and 500 genotypes in the UMN and NDSU breeding program (Figure 6), with no plateau reached for some traits. This indicates that training set size depends on the genetic architecture of the trait and that training set size was certainly a limiting factor in the URSN population (*n* = 222). Norman et al. (2018) observed a plateau at around 7000 lines when performing GP in various traits in wheat.

### 4.3 On the use of historical trials in breeding programs

Multiple studies have shown that using a less related population as a training set would probably reduce the predictive ability, compared to a training set within the same population and cohort (Adeyemo et al., 2023; Norman et al., 2018; J. Rutkoski et al., 2015). That situation requires constantly updating the training set with more phenotyping. Since phenotypic data from the URSN population are freely available, breeders could harness such a dataset to predict breeding values without any more phenotyping. Indeed, Rutkoski et al. (2015) showed that including a historical population in a training set could be useful in GP given that there are many and diverse genotypes with a high heritability. Adding the URSN lines to the full set of breeding lines led to similar predictive ability (Figure 5), in agreement with the majority of previous findings (Edwards et al., 2019). However, when the number of lines from the breeding population decreased, there was an advantage of adding the URSN lines (Figure 6), with a difference of predictive ability up to 0.19. This means that for the NDSU population and RKHS, the phenotyping effort could be reduced to 50 lines while having the same accuracy as when having 100 or 300 lines depending on the trait (Figure 6). This advantage is likely to be observed in other populations if the genetic relatedness is strong enough between the historical and the breeding population.

### 4.4 Usefulness of training set optimization with population size

Many algorithms have been developed to perform training set optimization for GP (Akdemir, 2018; Akdemir & Isidro‐Sánchez, 2019; Rincent et al., 2012). In this study, a new and more flexible algorithm was chosen with a SSI (Lopez‐Cruz & de los Campos, 2021; Lopez‐Cruz et al., 2020). The main features of this algorithm are (i) to provide a weighting for each individual selected in the training set, according to its closeness to the validation set, (ii) to select a training set specific to each genotype in the validation set, and (iii) to rapidly compute training set optimization and GP.

Training set size reduction and optimization were performed since the URSN population was composed of subpopulations originating from UMN and NDSU breeding lines. However, this did not always lead to a predictive ability increase (Figure 7B), and indeed, a small number of URSN lines were incorporated into the training set (Figure S12). Nevertheless, when using a reduced number of lines in the training set, adding URSN lines increased predictive ability (Figure 6). Training set optimization for the URN population led to more URSN genotypes being selected compared to UMN and NDSU breeding programs (Figure S12). This was expected given the closeness between URN and URSN populations compared to the other breeding programs. Otherwise, on average, a very small proportion of the optimized training set came from the URSN population.

## AUTHOR CONTRIBUTIONS

**Charlotte Brault**: Formal analysis; investigation; methodology; visualization; writing—original draft; writing—review and editing. **Emily J. Conley**: Data curation; formal analysis; validation; writing—review and editing. **Andrew J. Green**: Conceptualization; data curation; validation; writing—review and editing. **Karl D. Glover**: Data curation; formal analysis; validation; writing—review and editing. **Jason P. Cook**: Data curation; formal analysis; validation; writing—review and editing. **Harsimardeep S. Gill**: Investigation; validation; writing—review and editing. **Andrew C. Read**: Project administration; resources; writing—review and editing. **Jason D. Fiedler**: Conceptualization; funding acquisition; project administration; supervision; validation; writing—review and editing. **James A. Anderson**: Conceptualization; data curation; formal analysis; funding acquisition; project administration; supervision; writing—review and editing.

## CONFLICT OF INTEREST STATEMENT

The authors declare no conflicts of interest.

## Supporting information



Figure S1: URN design of experiment.
Figure S2: Number of genotypes and the first year of phenotyping.
Figure S3: Genomic imputation accuracy.
Figure S4: Genetic relatedness among organizations.
Figure S5: Genetic structure among organizations, result of PCA analysis.
Figure S6: Genetic relatedness between URSN and breeding programs, results of PCA analysis.
Figure S7: Comparison of rrBLUP and RKHS predictive ability.
Figure S8: Comparison of seven methods on predictive ability for the 3K genotypic array.
Figure S9: Comparison of seven methods on predictive ability for the 90K genotypic array.
Figure S10: URSN RKHS genomic prediction observed versus predicted.
Figure S11: Comparison of methods RR‐BLUP and RKHS method.
Figure S12: Optimized training set size and composition.
Table S1: Summary of fitting information.
Methods S1: GBS genotyping for the UMN population
Methods S2: Genomic imputation in the URSN population

## Data Availability

Phenotypic data are freely available on the T3 database (https://wheat.triticeaetoolbox.org/). All scripts, initial data, and output tables are available on Dryad: https://doi.org/10.5061/dryad.fj6q5743r. Supporting Information contains figures, tables, and methods that give further information and complementary results.
