# Supplementary material for: Leveraging historical trials to predict Fusarium head blight resistance in spring wheat breeding programs
Source: Plant Genome. 2025 Feb 6;18(1):e20559. doi: 10.1002/tpg2.20559 (PMC11800066; doi:10.1002/tpg2.20559)
Supplement: Supplementary file 1 [file TPG2-18-e20559-s001.pdf]

Leveraging historical disease trials to predict Fusarium head blight resistance in wheat breeding programs

Supplemental Material

Charlotte Brault[1,*], Emily J. Conley[1], Andrew J. Green[2], Karl D. Glover[3], Jason P. Cook[4], Harsimardeep S. Gill[1], Andrew C. Read[5], Jason D. Fiedler[6,*], and James A. Anderson [1]

[1]Department of Agronomy and Plant Genetics, University of Minnesota, Saint Paul, MN, USA

[2]Department of Plant Sciences, North Dakota State University, Fargo, ND, USA

[3]Agronomy, Horticulture, and Plant Science Department, South Dakota State University, Brookings, South Dakota, USA

[4]Plant Sciences and Plant Pathology Department, Montana State University, Bozeman, MT 59717, USA

[5]USDA-ARS, Plant Science Research Unit, St. Paul, Minnesota, USA

[6]USDA-ARS Cereal Crops Research Unit, Edward T. Schafer Agricultural Research Center, Fargo, ND, USA

[*]Corresponding authors: charlotte.brault@live.com; jason.fiedler@usda.gov

List of Figures

Figure S1 URN Design of experiment . . . 3
Figure S2 Number of genotypes and the first year of phenotyping . . . 4
Figure S3 Genomic imputation accuracy . . . 7
Figure S4 Genetic relatedness among organizations. . . . 8
Figure S5 Genetic structure among organizations, result of PCA analysis. . . . 9
Figure S6 Genetic relatedness between URSN and breeding programs, results of PCA analysis. . . . 10
Figure S7 Comparison of rrBLUP and RKHS predictive ability . . . 11
Figure S8 Comparison of seven methods on predictive ability for the 3K genotypic array . . . 12
Figure S9 Comparison of seven methods on predictive ability for the 90K genotypic array . . . 13
Figure S10 URSN RKHS genomic prediction observed vs. predicted . . . 14
Figure S11 Comparison of methods RR-BLUP and RKHS method . . . 14
Figure S12 Optimized training set size and composition . . . 15

List of Tables

Table S1 Summary of fitting information. . . . 4

List of methods

Methods S1 **5**

Methods S2 **6**

References **16**

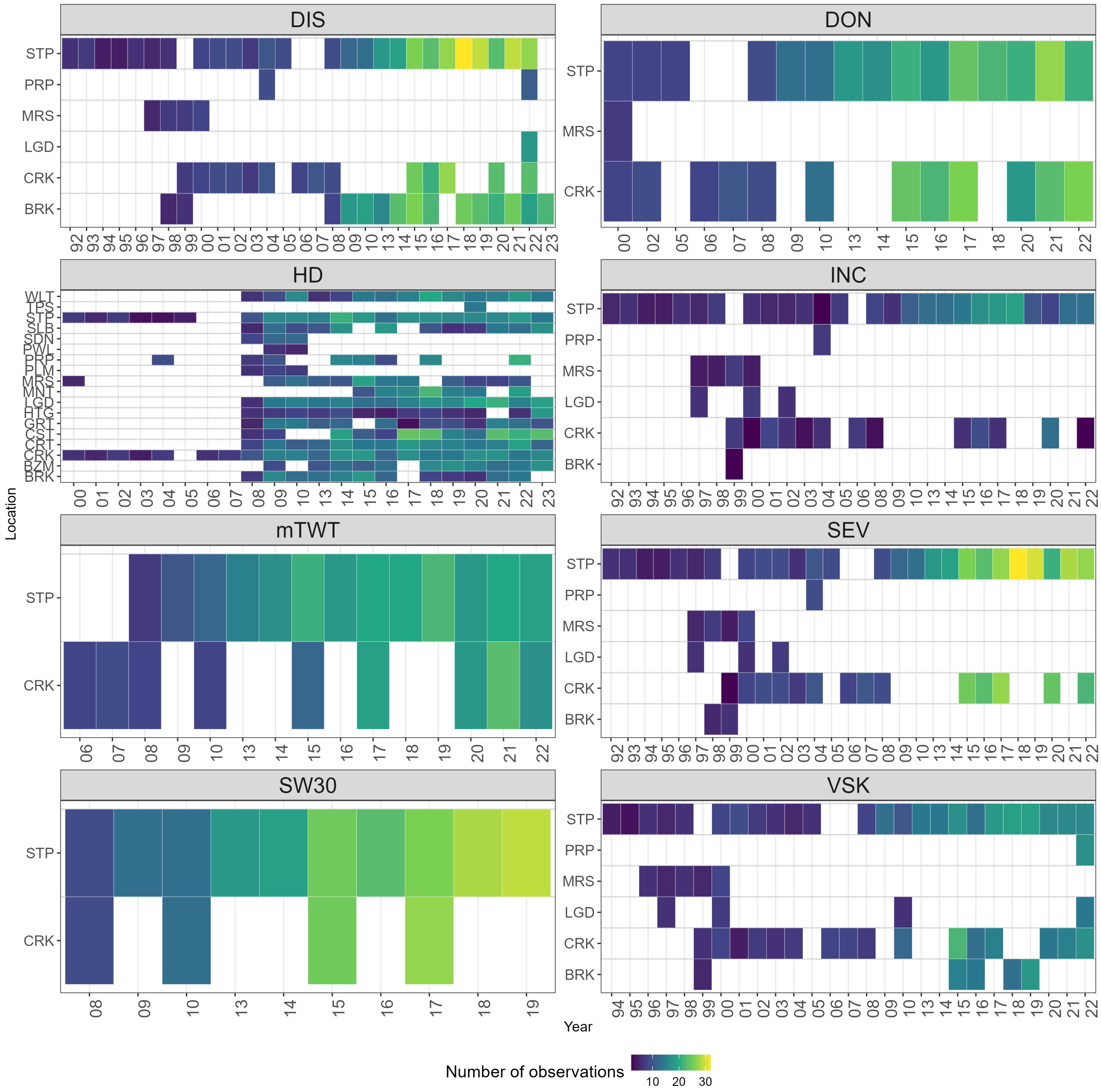

Figure S1: Number of genotypes scored for each environment and trait in the Uniform Regional Nursery (URN) population.

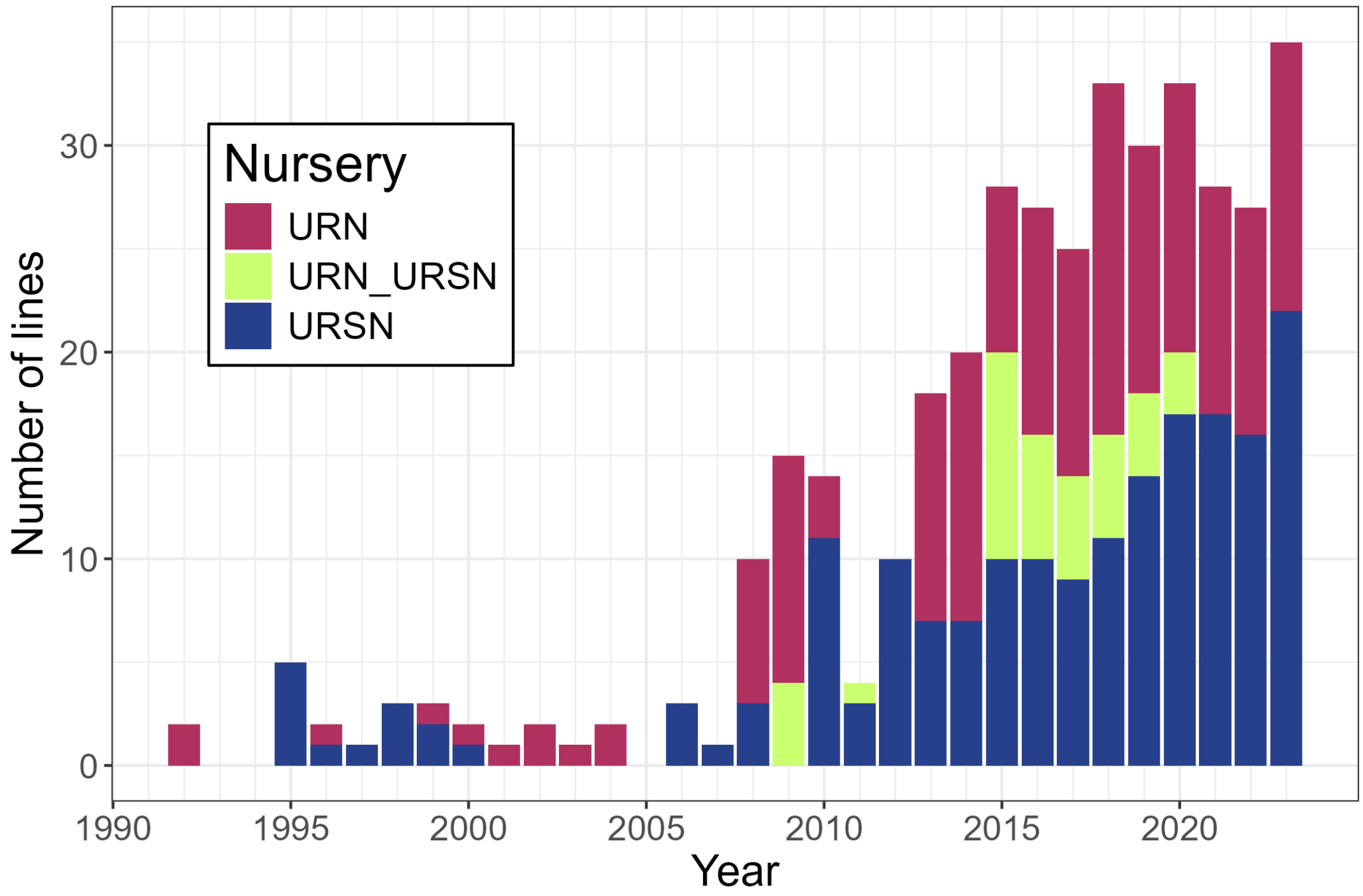

Figure S2: Number of genotypes and the first year of phenotyping for the two nurseries studied. URN: uniform regional nursery; URSN: uniform regional scab nursery.

Table S1: Summary of fitting information.

CO_trait: crop ontology trait code; missing.dat: percentage of missing data; ngeno: number of genotypes; var.geno: genetic variance; var.resid: residual variance, var.tot: total variance

URSN										
trait	CO_trait	miss.perc	ngeno	min	max	mean	var.geno	var.resid	var.tot	reliability
DIS	CO_321:0501030	24	222	0.2	99.0	27.7	67.370	122.327	189.698	0.646
DON	CO_321:0001154	51	210	0.2	75.1	8.0	7.672	30.062	37.734	0.370
HD	CO_321:0001233	57	211	155.0	204.0	184.2	4.890	1.440	6.329	0.877
INC	CO_321:0001149	26	222	0.5	100.0	82.4	24.843	103.253	128.096	0.438
MTWT	CO_321:0501106	68	203	7.0	12.8	11.0	0.222	0.189	0.411	0.673
SEV	CO_321:0001440	20	222	0.5	99.0	29.4	57.440	115.310	172.750	0.637
SW30	CO_321:0501083	80	129	1.7	37.4	18.0	12.374	7.264	19.638	0.732
VSK	CO_321:0001155	17	220	0.7	90.0	24.9	61.983	93.638	155.621	0.702

Methods S1: GBS genotyping for the UMN population

For the 2020-2023 UMN lines, single nucleotide polymorphism (SNP) markers were identified using genotyping-by-sequencing (GBS; Elshire et al., 2011). GBS libraries were constructed with a two-enzyme method as outlined by Poland et al. (2012), incorporating two modifications: 1) two unique barcodes were ligated to each sample to minimize sequencing bias of certain barcodes, and 2) the concentrations of barcodes and common adapters were increased to 0.1 μM and 50 μM, respectively. Each year, four-five libraries at 768-plex each were sequenced on an Illumina NovaSeq S1 lane to produce single-end 100 bp reads. Additionally each year, a subset of approximately 200 lines selected based on proportional pedigree relationship to the full breeding population, and their parents, were sequenced in a separate NovaSeq S1 lane at 250-plex to get higher sequencing coverage.

Raw sequencing reads were demultiplexed using Sabre (https://github.com/najoshi/sabre). Adapters and low-quality bases ($Q < 30$) were trimmed using Cutadapt (Martin, 2011). The trimmed reads were then aligned to the T. aestivum cv. Chinese Spring reference assembly, RefSeq v2.1 (Zhu et al., 2021) using the BWA-MEM algorithm with default parameters (Li, 2013). The aligned reads were filtered ($MAPQ < 40$) and sorted with SAMtools, and SNP calling was performed using BCFtools (Li, 2011; Li et al., 2009). Genotypes within a site were set to missing data based on read depth ($DP < 3$). At 768-plex, the DP3 filter removes a large number of sites. To compensate, sites were called using the higher coverage 250-plex lanes to obtain a list of true SNP sites. Then, any of those sites in the 768-plex lanes that were filtered based on $DP < 3$ were pushed back into the dataset, and monomorphic and multiallelic sites were filtered. Each year's dataset was imported and merged in TASSEL 5 (Bradbury et al., 2007). The 2020-2023 UMN merged dataset was additionally filtered, discarding sites with $\geq 20\%$ missing data, heterozygosity $> 10\%$, or $MAF < 0.01$.

Methods S2: Genomic imputation in the URSN population

The individuals from the URSN population, both genotyped with the 3K and the 90K array were used to estimate the imputation accuracy.

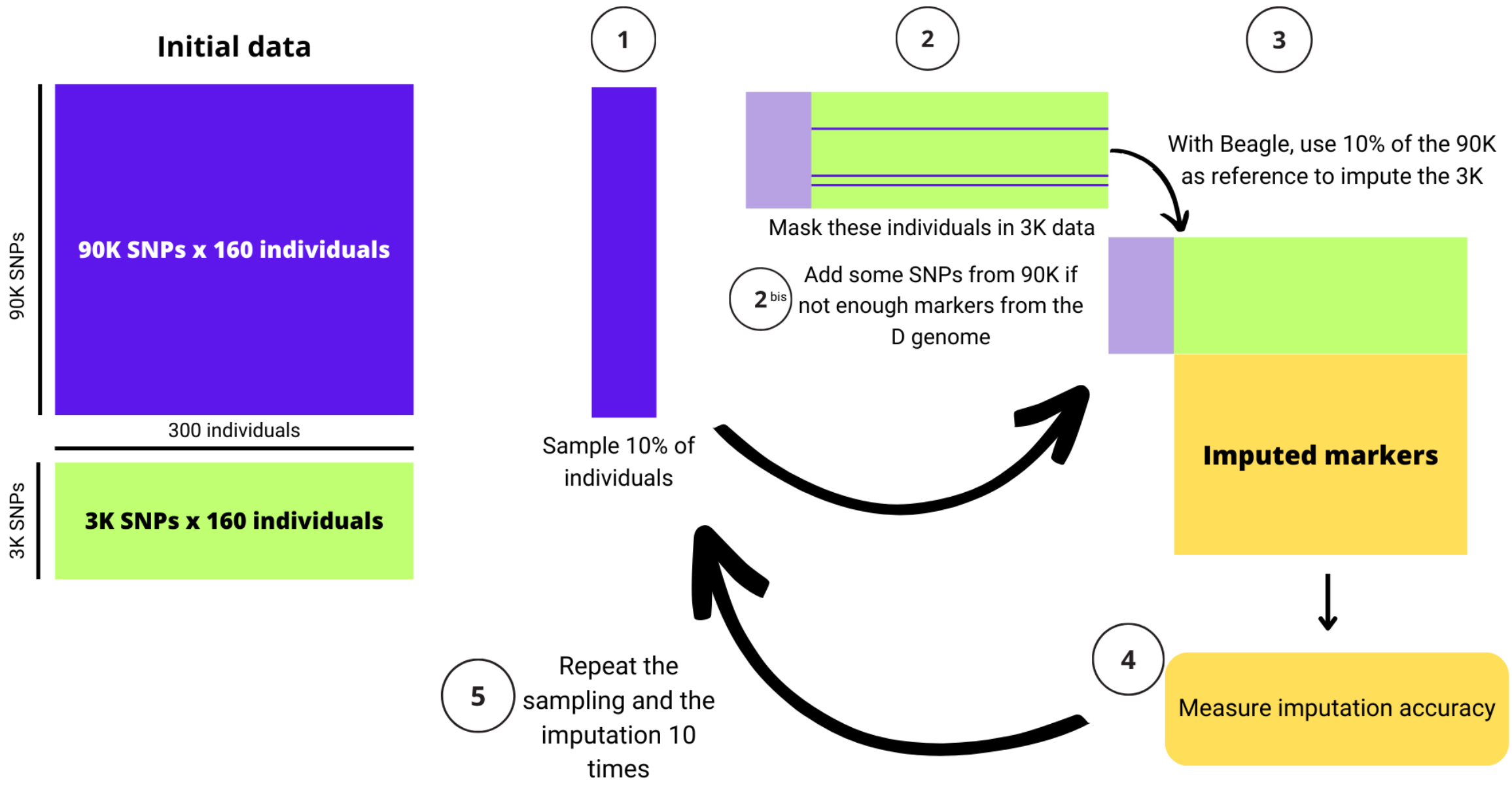

Description of the genomic imputation procedure

1. Sample 10% of the individuals from the 90K array
2. Mask these individuals in the 3K data, add a few more markers from the wheat D genome in order to garantee the imputation working
3. Using Beagle (v5.4) (Browning et al., 2018, 2021), use the 10% of the 90K individuals to impute the remaining 90% from 3K to 90K.
4. For each SNP and all imputed genotypes, generate a confusion matrix and estimate the accuracy as the ratio between the sum of the true positive and true negative, and the sum of the positive and negatives.
5. Repeat the sample and imputation procedure 10 times and aggregate the results

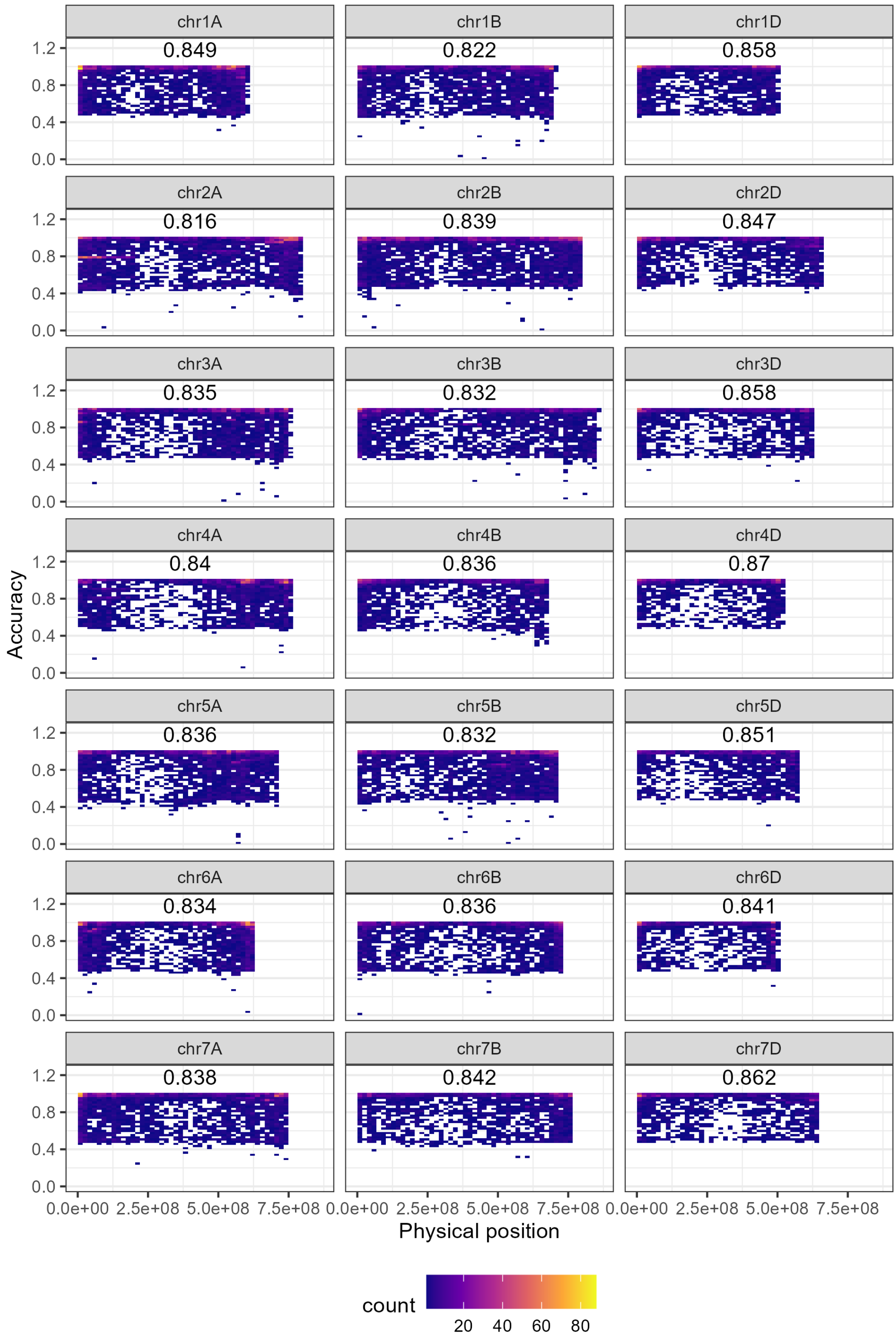

Figure S3: Genomic imputation accuracy in the URSN population. Imputation procedure described in Methods S2

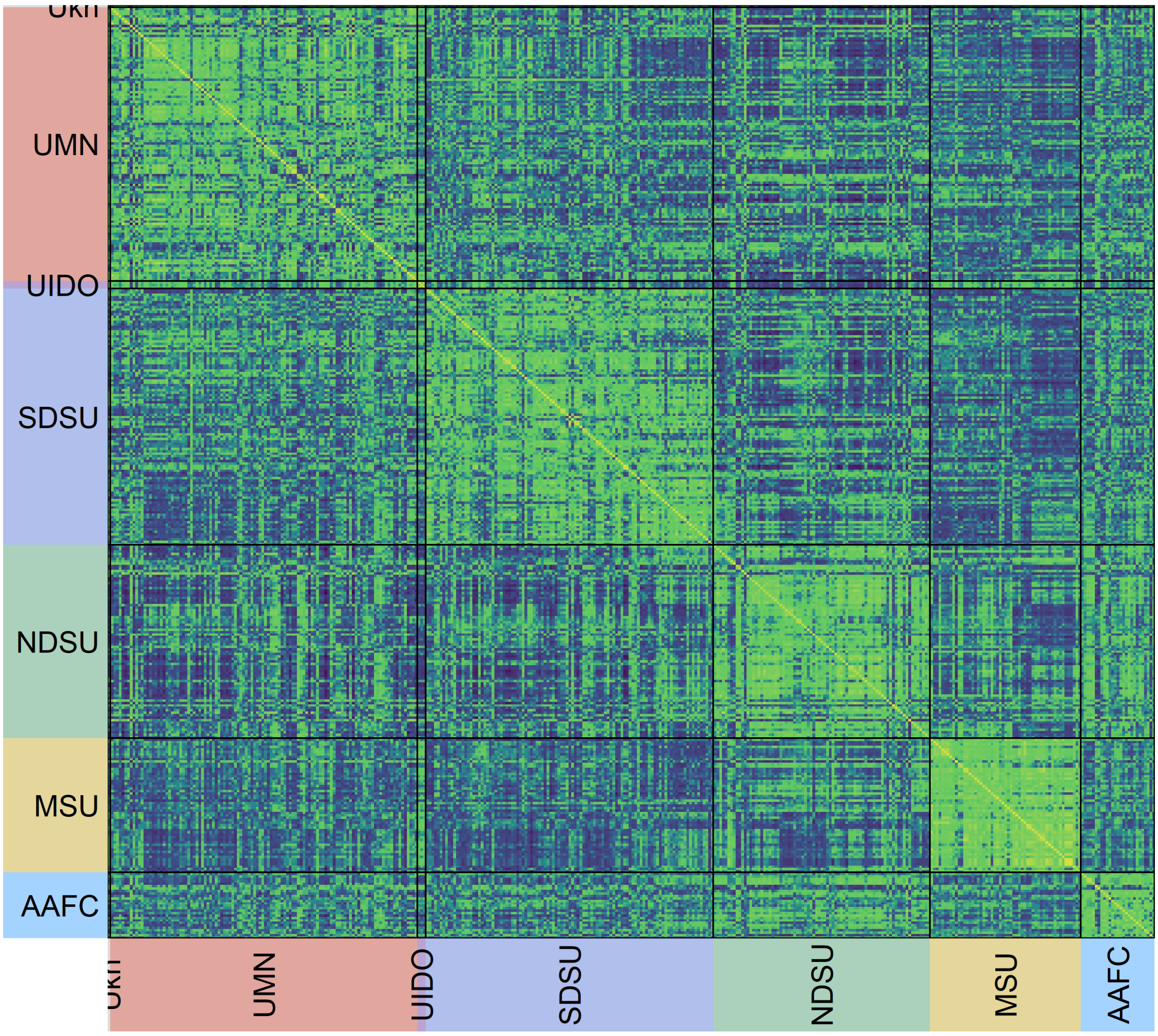

Figure S4: Additive genetic relatedness among genotypes, from the 3K genotypic array for the URSN population. The value on the top of each box corresponds to the average accuracy for each chromosome.

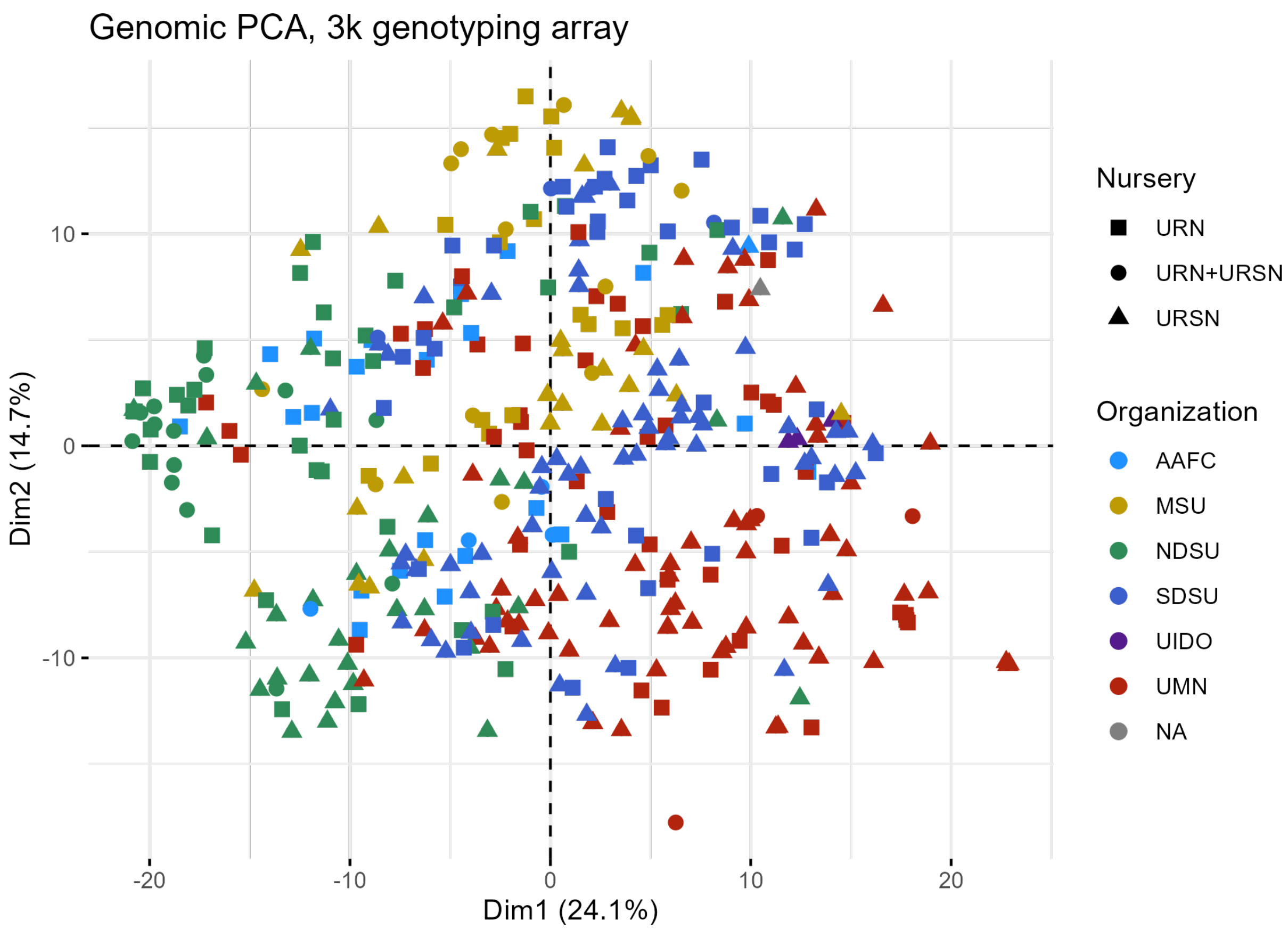

Figure S5: Genetic relatedness among organizations, results of a principal component analysis (PCA) projected on the first two axes, from the 3K genotypic array for the URSN population

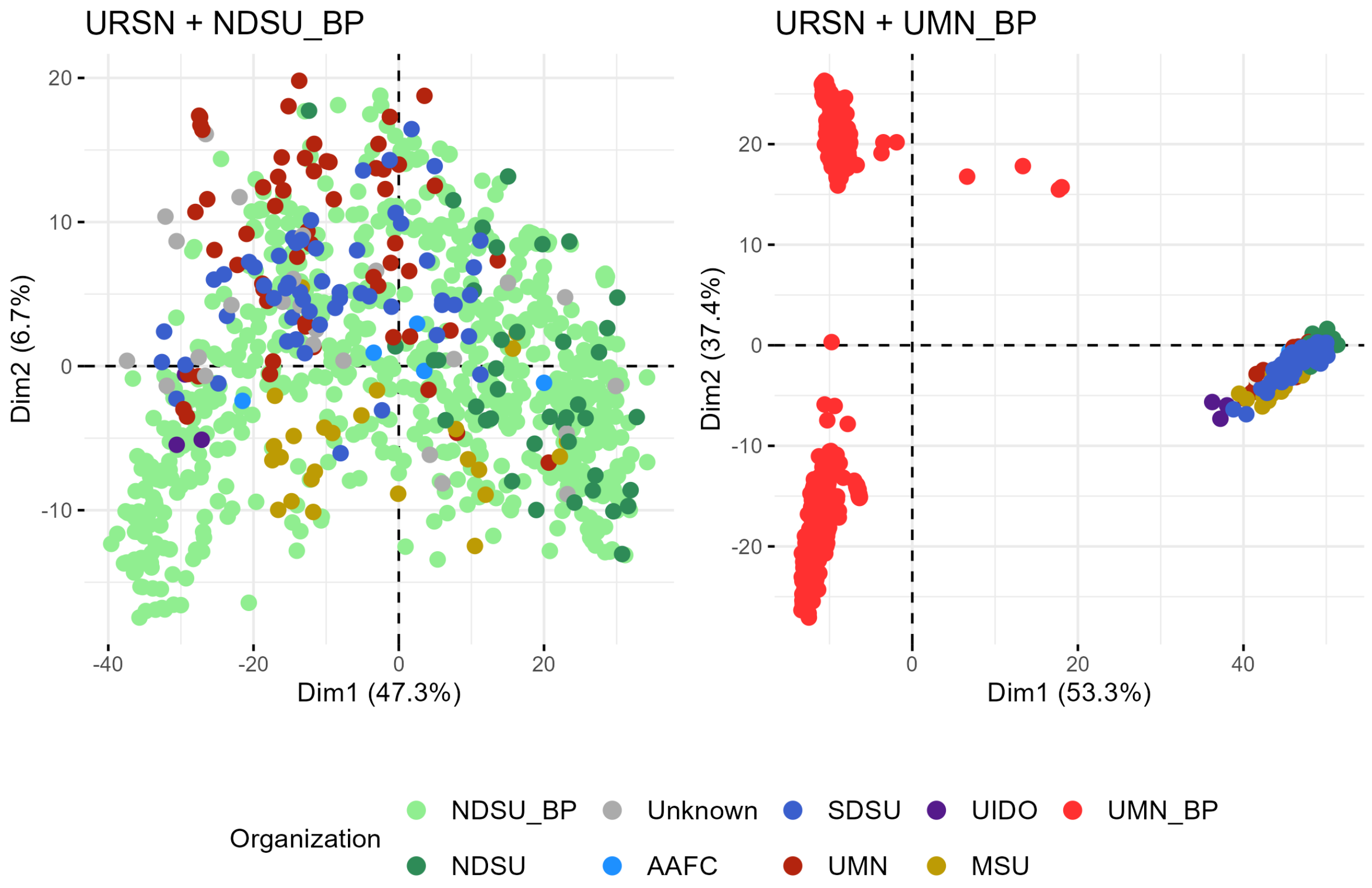

Figure S6: Genetic relatedness between URSN and breeding programs, results of PCA analysis. UMN BP: UMN breeding program (in red), NDSU BP: NDSU breeding program (in light green).

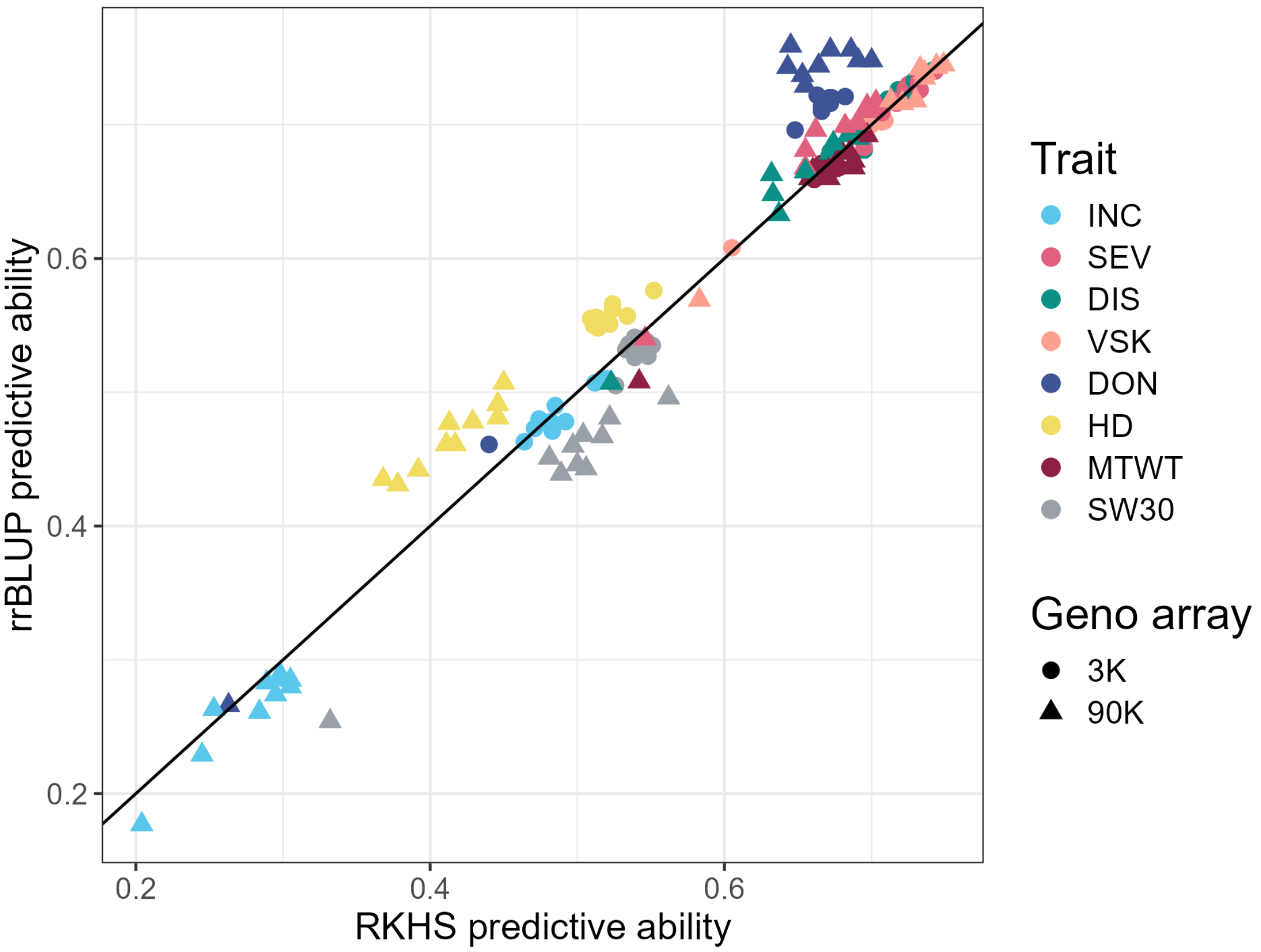

Figure S7: Comparison of rrBLUP and RKHS predictive ability across the eight traits for the two genotypic arrays. Predictive ability values are displayed for each cross-validation repetition (n=10)

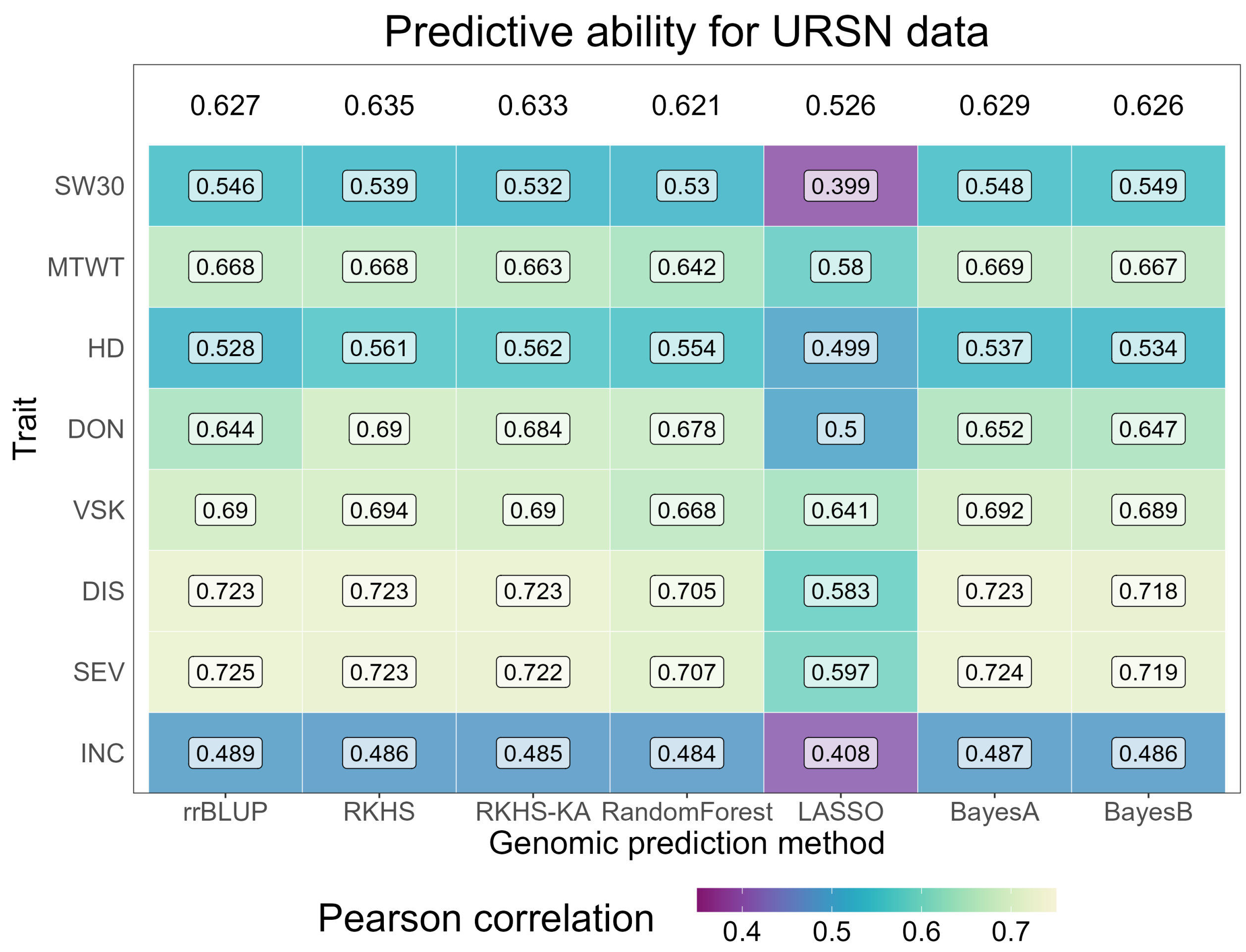

Figure S8: Comparison of genomic prediction (GP) methods on the uniform regional scab nursery (URSN) population for the 3K genotypic array (1999 SNPs and 222 genotypes) on eight traits. Average accuracy by method is indicated on top of the heatmap.

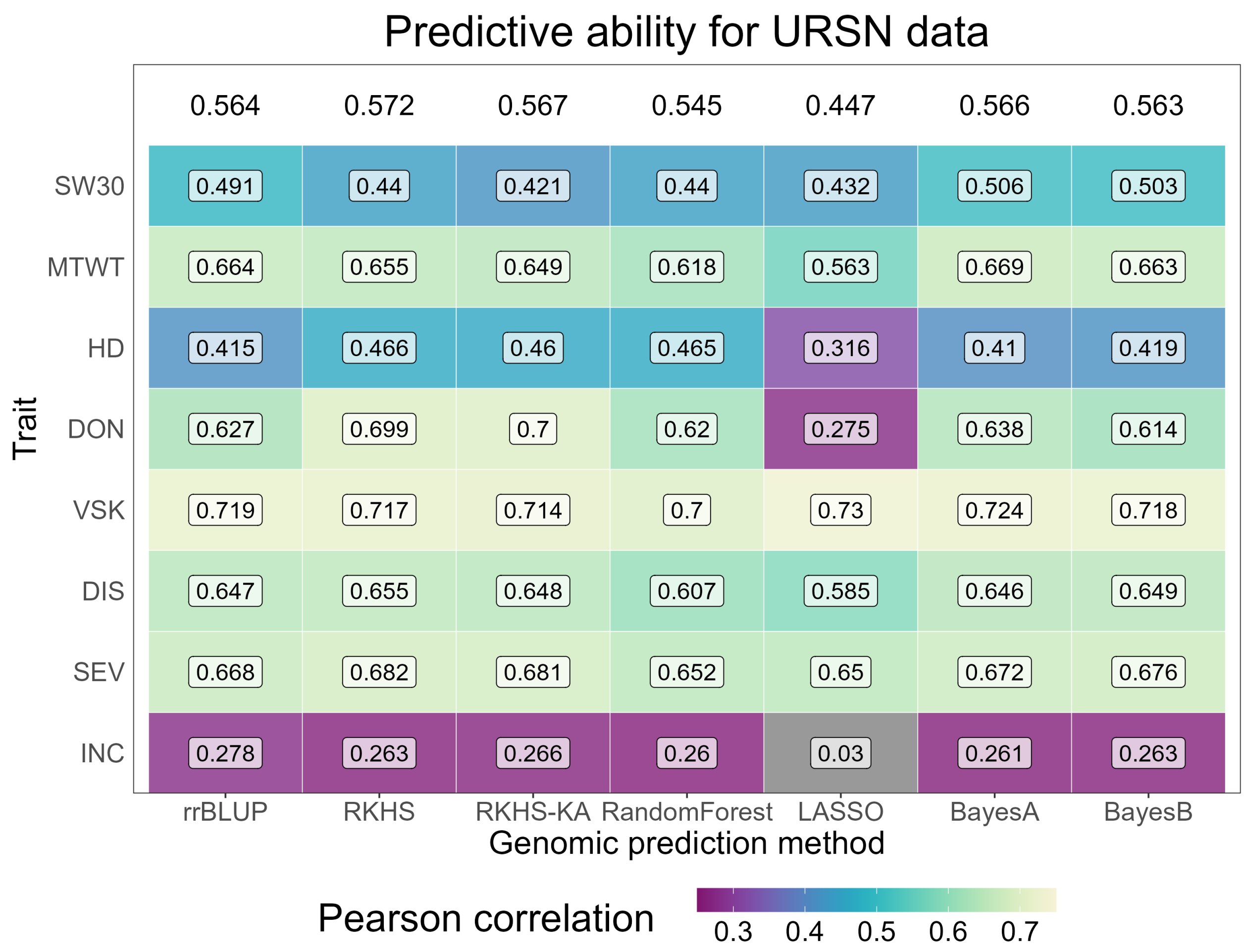

Figure S9: Comparison of genomic prediction (GP) methods on the uniform regional scab nursery (URSN) population for the 90K genotypic array (39,085 SNPs and 161 genotypes) on eight traits. Average accuracy by method is indicated on top of the heatmap.

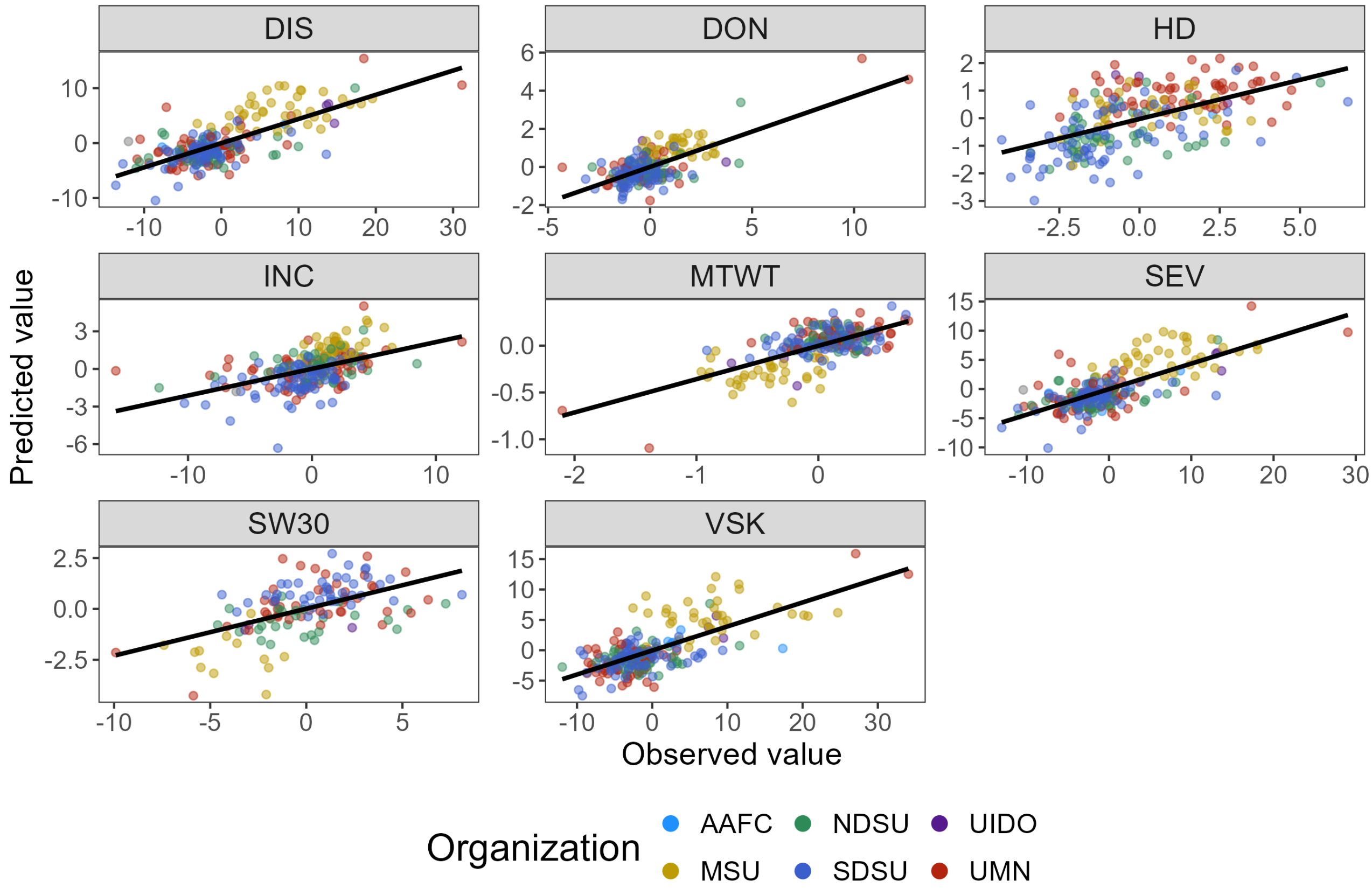

Figure S10: URSN genomic prediction observed vs. (mean) predicted genotypic value using the 3K genotypic array for 8 traits and RKHS method. The black line is the linear regression between observed and predicted genotypic value.

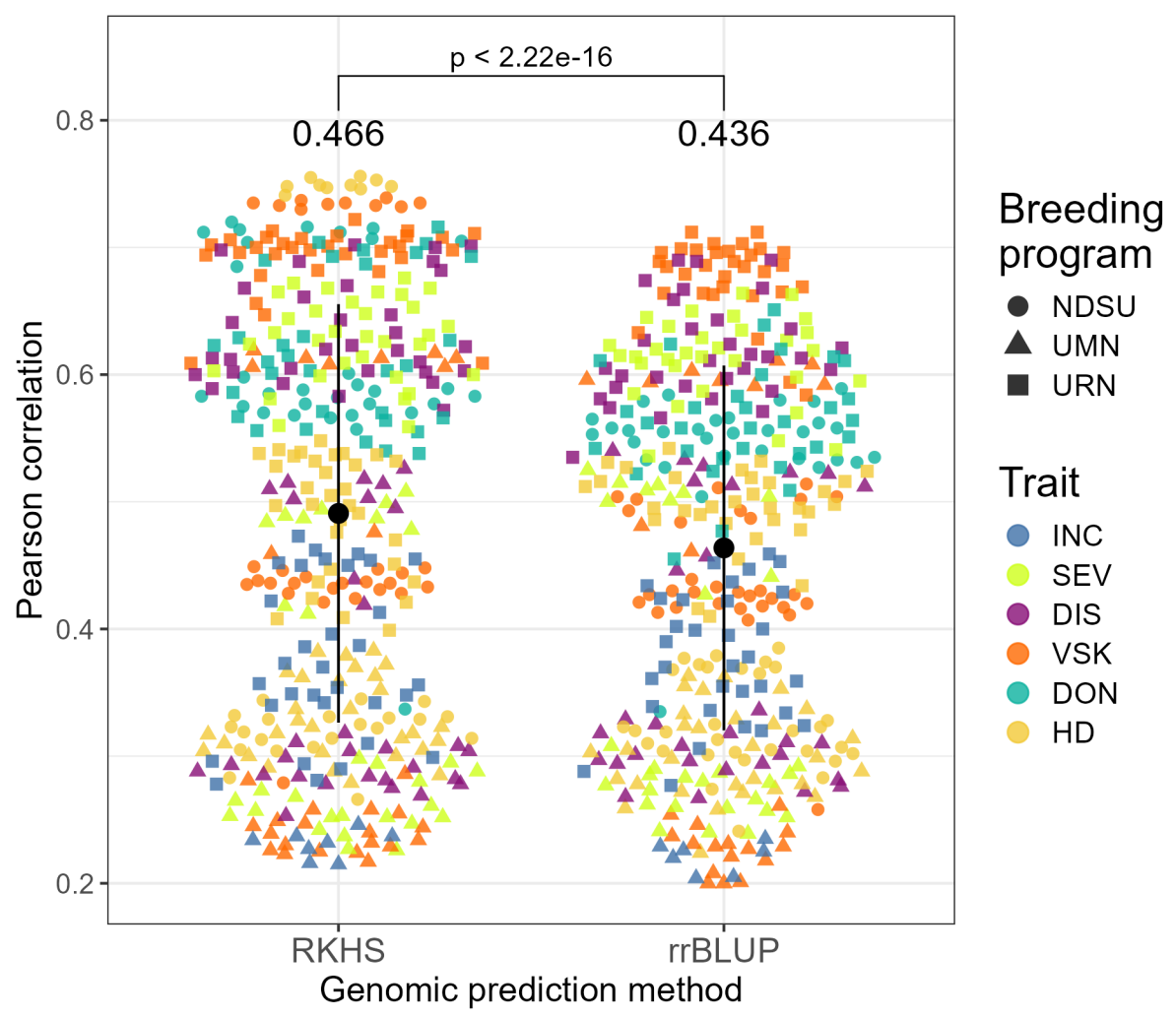

Figure S11: Comparison between RR-BLUP and RKHS genomic prediction methods when predicting breeding programs. Test for significant difference was done using a t-test, the black point and lines represent the mean and the standard deviation.

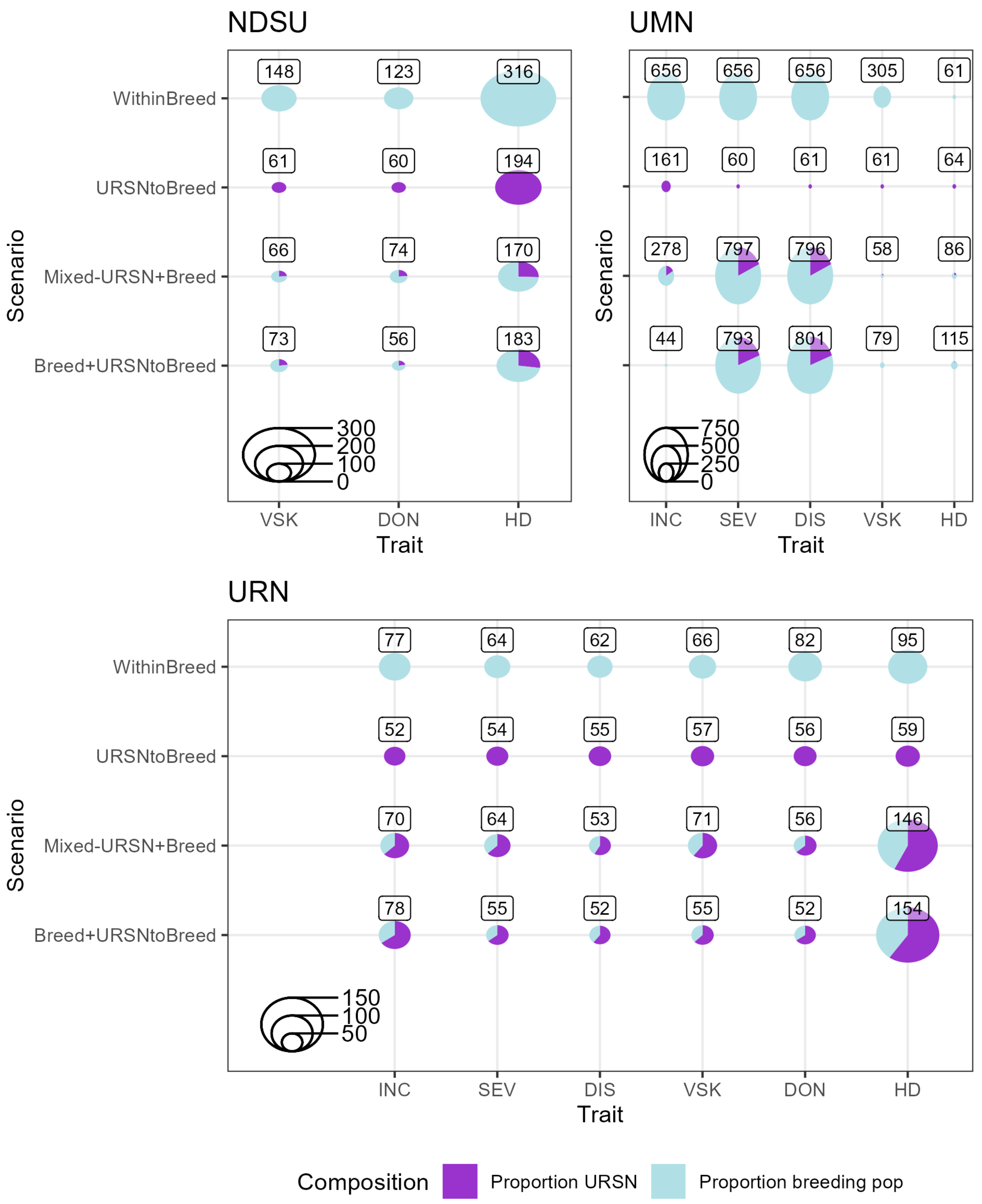

Figure S12: Training set size and composition after training set optimization by sparse selection index (SSI) for the 3 breeding populations, the 6 traits and 4 scenarios.

References

Bradbury, P. J., Zhang, Z., Kroon, D. E., Casstevens, T. M., Ramdoss, Y., & Buckler, E. S. (2007). TASSEL: Software for association mapping of complex traits in diverse samples. *Bioinformatics*, *23*(19), 2633–2635. https://doi.org/10.1093/bioinformatics/btm308

Browning, B. L., Tian, X., Zhou, Y., & Browning, S. R. (2021). Fast two-stage phasing of large-scale sequence data. *The American Journal of Human Genetics*, *108*(10), 1880–1890. https://doi.org/10.1016/j.ajhg.2021.08.005

Browning, B. L., Zhou, Y., & Browning, S. R. (2018). A One-Penny Imputed Genome from Next-Generation Reference Panels. *The American Journal of Human Genetics*, *103*(3), 338–348. https://doi.org/10.1016/j.ajhg.2018.07.015

Elshire, R. J., Glaubitz, J. C., Sun, Q., Poland, J. A., Kawamoto, K., Buckler, E. S., & Mitchell, S. E. (2011). A Robust, Simple Genotyping-by-Sequencing (GBS) Approach for High Diversity Species. *PLOS ONE*, *6*(5), e19379. https://doi.org/10.1371/journal.pone.0019379

Li, H. (2011). A statistical framework for SNP calling, mutation discovery, association mapping and population genetical parameter estimation from sequencing data. *Bioinformatics*, *27*(21), 2987–2993. https://doi.org/10.1093/bioinformatics/btr509

Li, H. (2013, May 26). *Aligning sequence reads, clone sequences and assembly contigs with BWA-MEM*. arXiv: 1303.3997 [q-bio]. https://doi.org/10.48550/arXiv.1303.3997

Li, H., Handsaker, B., Wysoker, A., Fennell, T., Ruan, J., Homer, N., Marth, G., Abecasis, G., Durbin, R., & 1000 Genome Project Data Processing Subgroup. (2009). The Sequence Alignment/Map format and SAMtools. *Bioinformatics*, *25*(16), 2078–2079. https://doi.org/10.1093/bioinformatics/btp352

Martin, M. (2011). Cutadapt removes adapter sequences from high-throughput sequencing reads. *EMBnet.journal*, *17*(1), 10–12. https://doi.org/10.14806/ej.17.1.200

Poland, J. A., Brown, P. J., Sorrells, M. E., & Jannink, J.-L. (2012). Development of High-Density Genetic Maps for Barley and Wheat Using a Novel Two-Enzyme Genotyping-by-Sequencing Approach. *PLOS ONE*, *7*(2), e32253. https://doi.org/10.1371/journal.pone.0032253

Zhu, T., Wang, L., Rimbert, H., Rodriguez, J. C., Deal, K. R., De Oliveira, R., Choulet, F., Keeble-Gagnère, G., Tibbits, J., Rogers, J., Eversole, K., Appels, R., Gu, Y. Q., Mascher, M., Dvorak, J., & Luo, M.-C. (2021). Optical maps refine the bread wheat

Triticum aestivum cv. Chinese Spring genome assembly. *The Plant Journal*, *107*(1), 303–314. https://doi.org/10.1111/tpj.15289
